# Supplementary material for: Drivers of global mangrove loss and gain in social-ecological systems
Source: Nat Commun. 2022 Oct 26;13:6373. doi: 10.1038/s41467-022-33962-x (PMC9606261; doi:10.1038/s41467-022-33962-x)
Supplement: Supplementary file 1 — Supplementary Information [file 41467_2022_33962_MOESM1_ESM.pdf]

Drivers of global mangrove loss and gain in social-ecological systems

Supplementary Figures

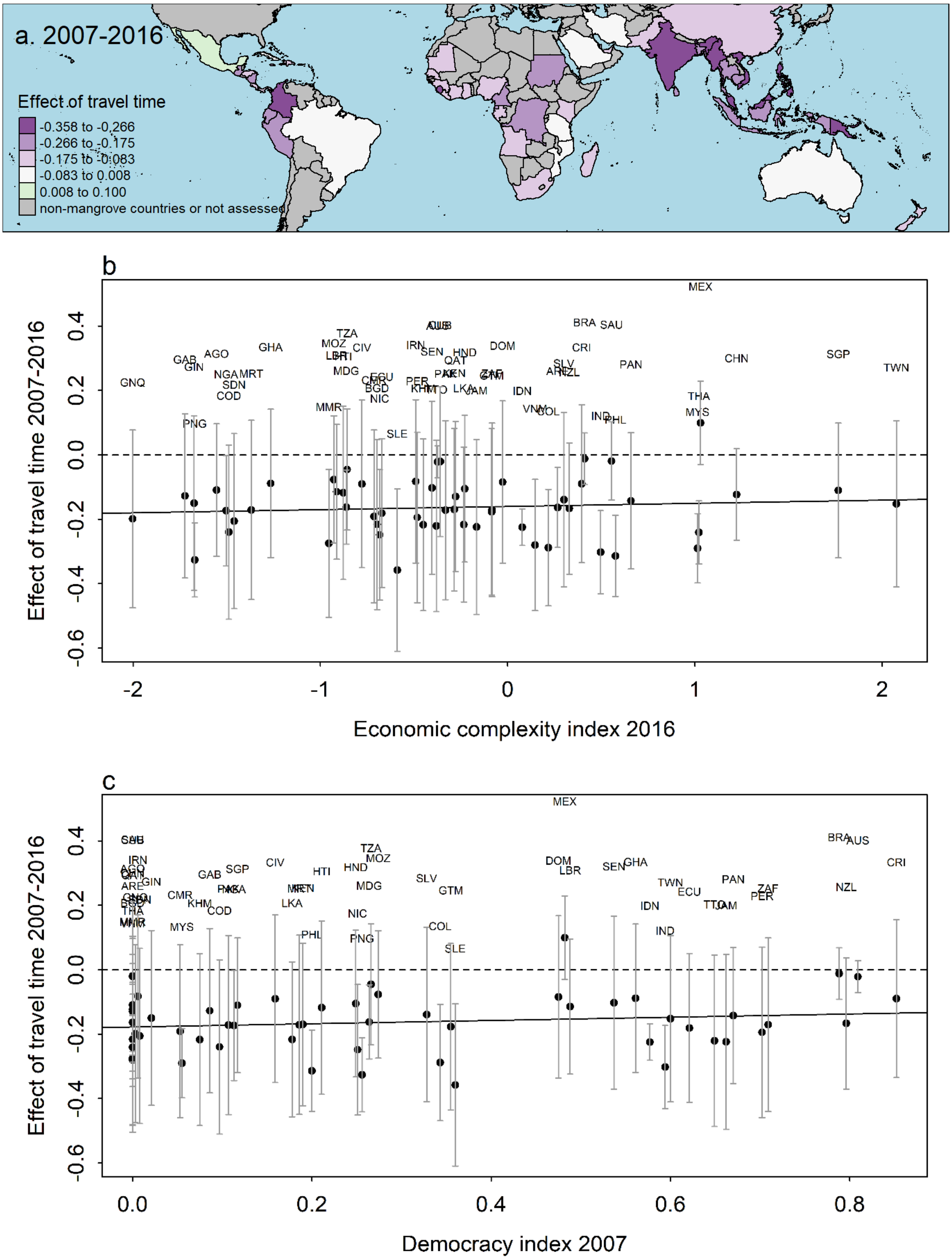

Supplementary Fig. 1. a, effect of travel time to nearest city per country on percent net loss 2007-2016. Effects are the country random slope estimates from the mixed-effects model (n = 56 countries). **b,c,** Influence of a country's economic complexity index (b) and democracy index (c) on the effect of travel time to nearest city on percent net loss of mangrove cover in

2007-2016. The y-axis points and error bars are the country random slope estimates and standard errors for travel time to nearest city from the mixed-effects model (n = 56 countries). The regression lines represent linear regression models between the slope estimate for travel time to nearest city and economic complexity index (b) and democracy index (c) for each country. Points are labelled by country code (ISO3C) offset above the point. In order from lowest to highest value these are: (a) GNQ = Equatorial Guinea, GAB = Gabon, GIN = Guinea, PNG = Papua New Guinea, AGO = Angola, NGA = Nigeria, COD = Congo, the Democratic Republic of the, SDN = Sudan, MRT = Mauritania, GHA = Ghana, MMR = Myanmar, MOZ = Mozambique, LBR = Liberia, HTI = Haiti, MDG = Madagascar, TZA = Tanzania, United Republic of, CIV = Côte d'Ivoire, CMR = Cameroon, BGD = Bangladesh, NIC = Nicaragua, ECU = Ecuador, SLE = Sierra Leone, IRN = Iran, Islamic Republic of, PER = Peru, KHM = Cambodia, SEN = Senegal, TTO = Trinidad and Tobago, AUS = Australia, CUB = Cuba, PAK = Pakistan, KEN = Kenya, QAT = Qatar, LKA = Sri Lanka, HND = Honduras, JAM = Jamaica, GTM = Guatemala, ZAF = South Africa, DOM = Dominican Republic, IDN = Indonesia, VNM = Viet Nam, COL = Colombia, ARE = United Arab Emirates, SLV = El Salvador, NZL = New Zealand, CRI = Costa Rica, BRA = Brazil, IND = India, SAU = Saudi Arabia, PHL = Philippines, PAN = Panama, MYS = Malaysia, THA = Thailand, MEX = Mexico, CHN = China, SGP = Singapore, TWN = Taiwan, Province of China, and (b) AGO, BGD, CHN, CUB, MMR, QAT, SAU, THA, ARE, VNM, GNQ, IRN, SDN, GIN, CMR, MYS, KHM, GAB, COD, PAK, NGA, SGP, CIV, LKA, MRT, KEN, PHL, HTI, HND, NIC, PNG, MDG, TZA, MOZ, SLV, COL, GTM, SLE, DOM, MEX, LBR, SEN, GHA, IDN, IND, TWN, ECU, TTO, JAM, PAN, PER, ZAF, BRA, NZL, AUS, CRI.

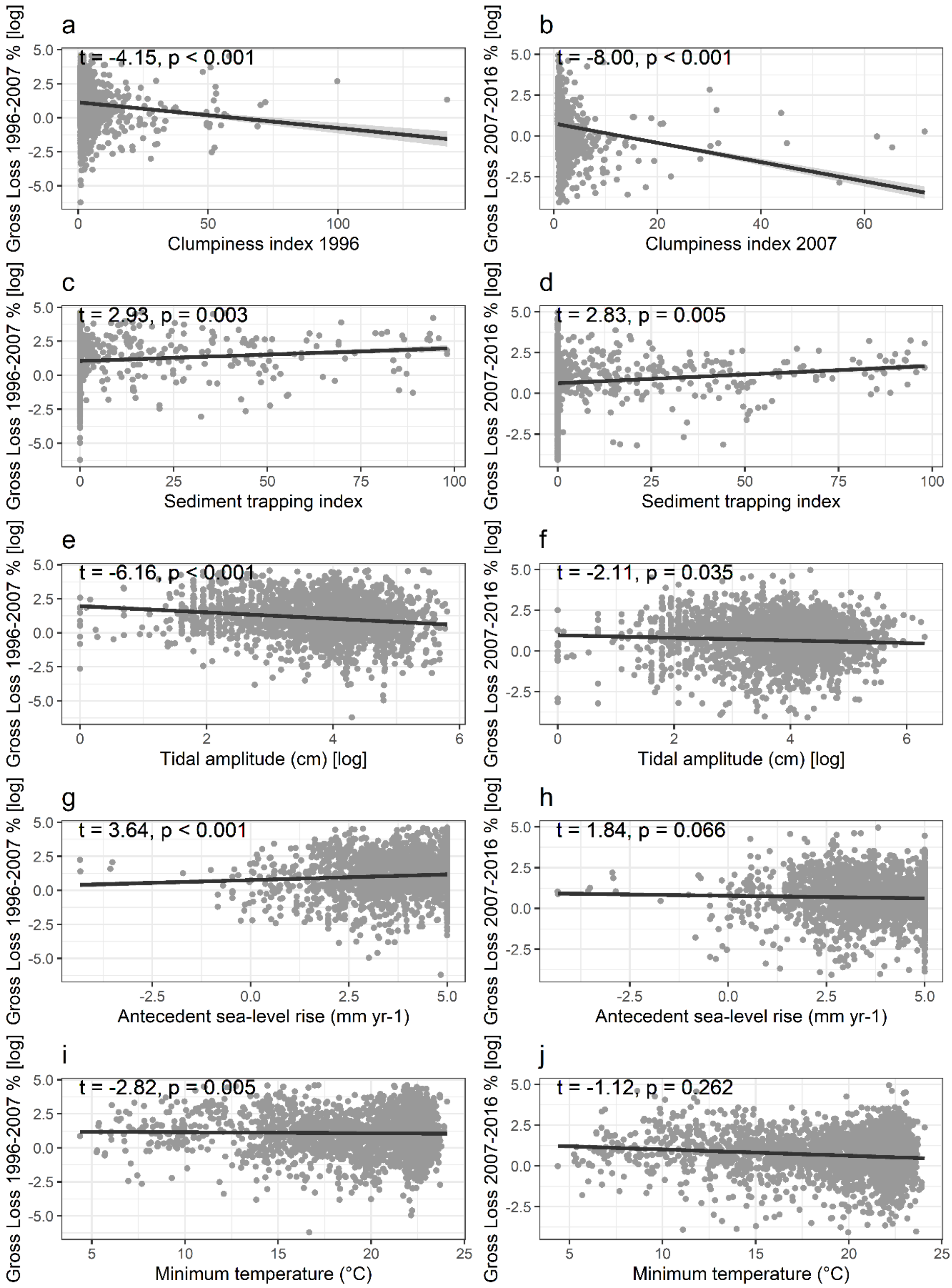

Supplementary Fig. 2. Biophysical drivers of mangrove gross loss. **a,b,** mangrove fragmentation and % gross loss 1996-2007 (a) and 2007-2016 (b). **c,d,** sediment availability and % gross loss 1996-2007 (c) and 2007-2016 (d). **e,f,** mean-weighted tidal amplitude and % gross loss 1996-2007 (e) and 2007-2016 (f). **g,h,** mean-weighted antecedent sea-level rise per year from 1993-2015 and % gross loss 1996-2007 (g) and 2007-2016 (h). **i,j,** mean minimum temperature of the coldest month from 1970-2000 and % gross loss 1996-2007 (i) and 2007-2016 (j). *n* = 2425 and 2637 mangrove geomorphic units for % gross loss 1996-2007 and 2007-2016, respectively. Shown are the fitted regression lines with 95% confidence intervals and t-values and p-values derived from two sample t-tests.

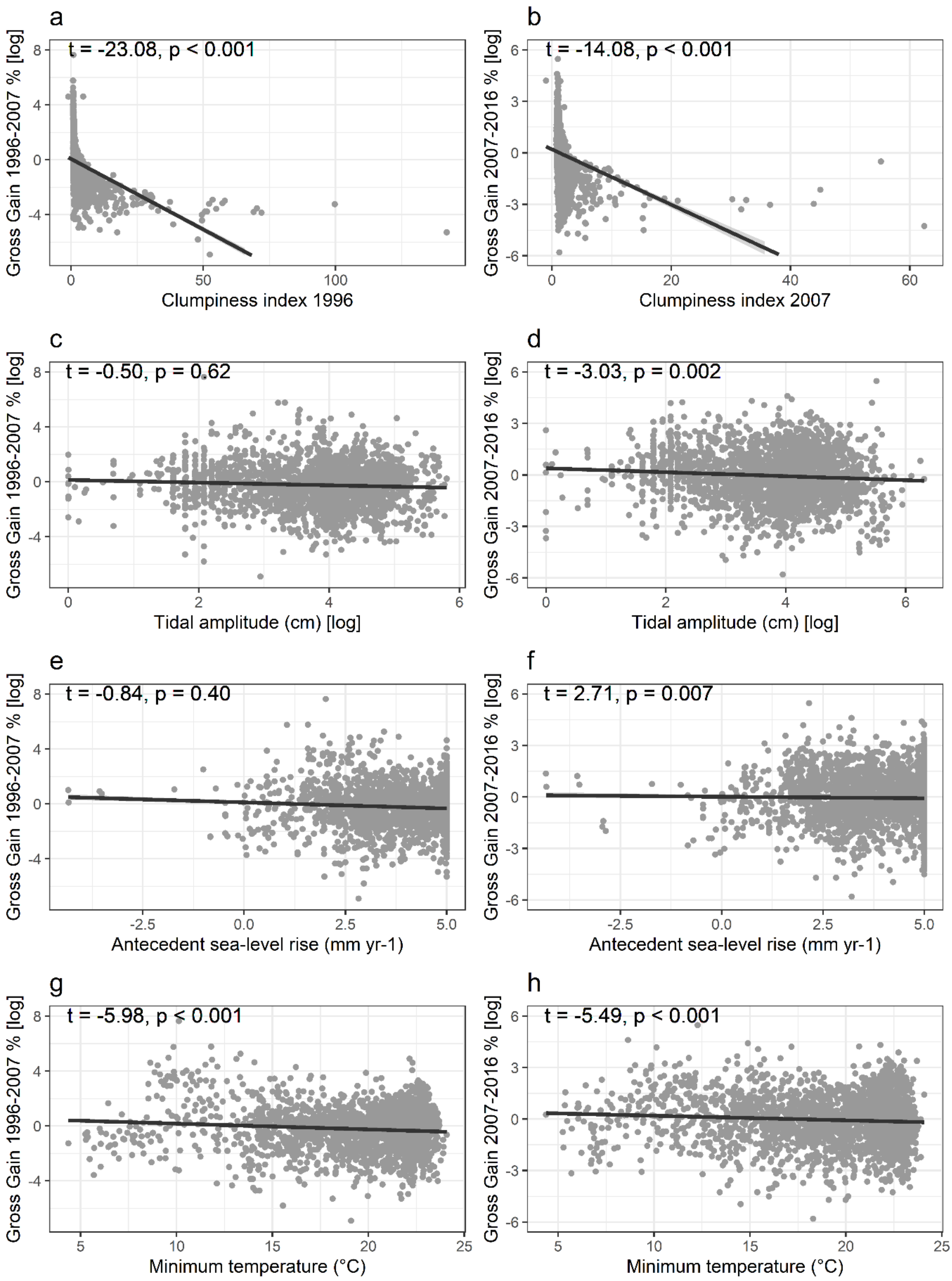

Supplementary Fig. 3. Biophysical drivers of mangrove gross gain. **a,b,** mangrove fragmentation and % gross gain 1996-2007 (a) and 2007-2016 (b). **c,d,** mean-weighted tidal amplitude and % gross gain 1996-2007 (c) and 2007-2016 (d). **e,f,** mean-weighted antecedent sea-level rise per year from 1993-2015 and % gross gain 1996-2007 (e) and 2007-2016 (f). **g,h,** mean minimum temperature of the coldest month from 1970-2000 and % gross gain 1996-2007 (g) and 2007-2016 (h). n = 2341 and 2554 mangrove geomorphic units for % gross gain 1996-2007 and 2007-2016, respectively. Shown are the fitted regression lines with 95% confidence intervals and t-values and p-values derived from two sample t-tests.

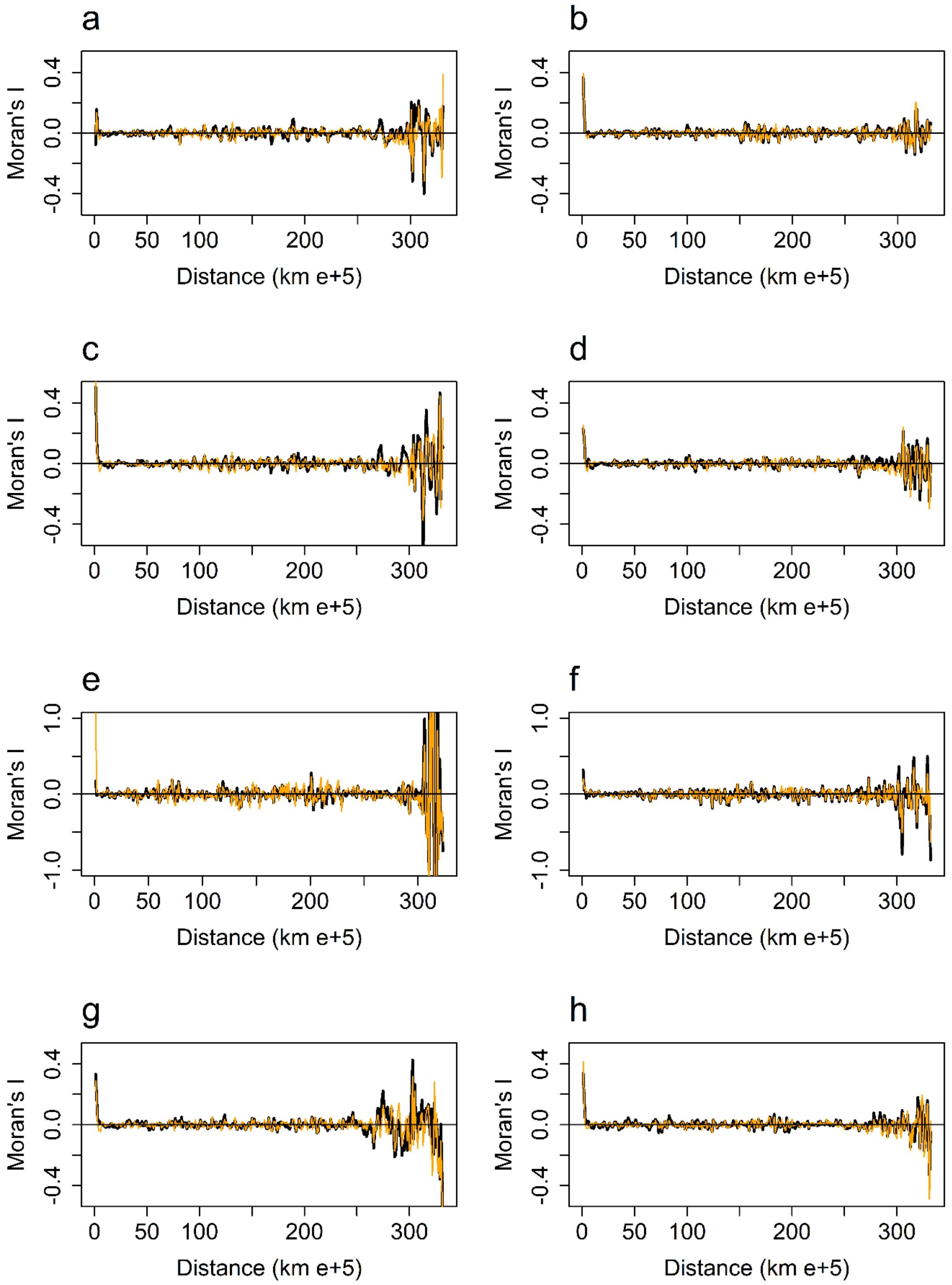

Supplementary Fig. 4. Correlograms for mangrove change indicators across time periods, comparing the adopted multi-level model (random coefficients model; black line) and simultaneous autoregressive model (yellow line). a, % net loss 1996-2007. b, % net loss 2007-2016. c, % gross loss 1996-2007. d, % gross loss 2007-2016. e, % net gain 1996-2007. f, % net gain 2007-2016. g, % gross gain 1996-2007. h, % gross gain 2007-2016.

Drivers of global mangrove loss and gain in social-ecological systems

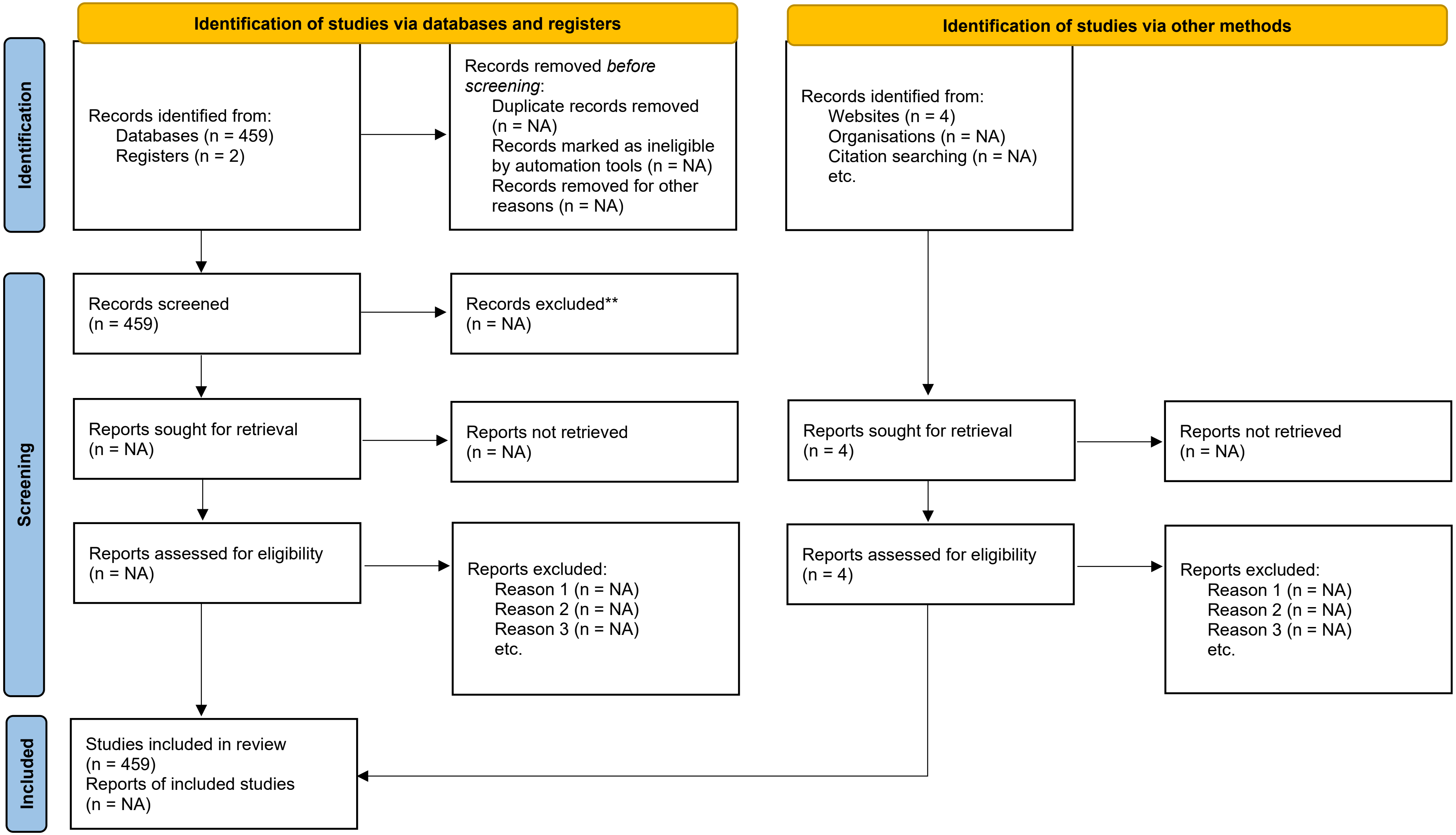

Supplementary Fig. 5. PRISMA flow diagram for Community Forestry systematic review

Supplementary Tables

Supplementary Table 1. Potential drivers of hotspots of mangrove loss, where loss at the geomorphic unit was two standard deviations more (high loss) or less (low loss) than the country average, respectively.

**Country and ISO3C**	**Geomorphic unit (Class and ID)**	**Region**	**Mangrove change variable**	**Likely reasons**	**Protected Area**
**HIGH LOSS**					
Belize BLZ	Lagoon 7955	North of Chetumal, Mar Caribe, Quintana Roo	Net Loss %<br>Gross Loss %	Urban and tourist development on the western side of the lagoon north of Chetumal	No
Brazil BRA	Open Coast 61427	Near border with French Guiana	Net Loss %<br>Gross Loss %	Mud waves associated with sediment transportation north of the mouth of the Amazon River.	Cabo Orange National Park
Colombia COL	Delta 8779	Barranquilla	Net Loss %<br>Gross Loss %	Agriculture (grazing land) in the non-protected areas. Possibly also dieback from drought associated with ENSO.	Partially - Sistema Delta Estuarino del Río Magdalena, Ciénaga Grande de Santa Marta Ramsar Site (International); Isla de Salamanca Park Way (national); Cienaga Grande de Santa Marta UNESCO-MAB Biosphere Reserve (International)
French Guiana GUF	Open Coast 61500	Sinnamary, Cayenne	Net Loss %<br>Gross Loss %	Agriculture – grazing land	Guyane Regional Nature Park (National); Estuaire du fleuve Sinnamary Ramsar Site (International)
Indonesia IDN	Estuary 3023	Luwu Timur, Sulawesi Selatan	Net Loss %	Extensive loss to aquaculture ponds.	No
Indonesia IDN	Open Coast 61296	Gorontalo, Sulawesi	Net Loss %<br>Gross Loss %	Extensive loss to aquaculture, particularly in PA with strictest protection.	Partially - Cagar Alam Darat (Nature Reserve)
Panama PAN	Open Coast 61769	Chame Bay Mangroves	Net Loss %<br>Gross Loss %	Aquaculture, agriculture, and coal production.	Bahía de Chame Muliple Use Areas (National)
Mexico MEX	Estuary 8228	Tampico, Veracruz, Gulf of Mexico	Net Loss %<br>Gross Loss %	Urban and rural expansion.	No
	Estuary 473				

Country and ISO3C	Geomorphic unit (Class and ID)	Region	Mangrove change variable	Likely reasons	Protected Area
Mozambique MOZ	Estuary 493<br>Estuary 497<br>Estuary 516	Nova Mambone	Gross Loss %	Natural hydrodynamic processes, possibly river erosion.	No
Mozambique MOZ	Estuary 640	Macuze	Gross Loss %	Agriculture – rice production	No
Pakistan PAK	Delta 8765	Keti Bunder	Gross Loss %	Aquaculture and agriculture.	Partially - Keti Bunder South Wildlife Sanctuary (National); Marho Kotri Wildlife Sanctuary (National)
Suriname SUR	Open Coast 62843	Wageningen, Groot Henar	Gross Loss %	Agriculture	Bigi Pan Multiple Use Area (national marine protected area)
Thailand THA	Delta 8748	Bay of Bangkok, Samut Prakan, Samut Sakhon, Samut Songkhran	Gross Loss %	Highly fragmented, agriculture and urban expansion.	No
Thailand THA	Open Coast 62072	Bay of Bangkok, Ban Laem	Gross Loss %	Extensive loss to aquaculture and agriculture. Settlements along river reaches.	Partially - Phetchaburi and Prachuap Khirikhan Environmental Protected Area (marine - national)
United States USA	Lagoon 20129	Key Largo and North Key Largo, Miami	Net Loss %<br>Gross Loss %	Highway to Key West and road passing through the wetland, urban expansion on peninsula.	Partially - Everglades National Park (national)
**LOW LOSS**					
Ecuador ECU	Delta 8762	Isla Puna	Net Loss %	Protected area	Partially. Manglares Churute Ecological Reserve, Manglares El Morro Wildlife Refuge, Manglares el Salado Fauna Protection Reserve
Ghana GHA	Delta 8830	Anloga, Keta	Net Loss %	Protected area	Anlo-Keta lagoon complex Ramsar Site (International)
Guyana GUY	Open Coast 61757	Near border with Venezuela	Gross Loss %	Mud waves associated with sediment transportation north of the mouth of the Amazon River.	Parque Nacional Delta del Orinoco

Supplementary Table 2. Potential drivers of hotspots of mangrove gain, where gain at the geomorphic unit was two standard deviations more (high gain) or less (low gain) than the country average, respectively.

Country and ISO3C	Geomorphic unit (Class and ID)	Region	Mangrove change variable	Likely reasons	Protected Area
**HIGH GAIN**					
Guinea GIN	Estuary 4737	Makira/Hamdalay, Forecariah Prefecture	Net Gain %	Low development pressure allows regeneration from natural hydrodynamic processes.	No
Micronesia, Federated States of FSM	Open Coast 61699	Danipei	Net Gain % Gross Gain %	Fairly stable – gain higher than average across 4 geomorphic units which are all stable.	No
Gambia GMB	Delta 8815	Foundiougne	Gross Gain %	Large, protected area allows regeneration from natural hydrodynamic processes.	Delta du Saloum National Park UNESCO-MAB Biosphere Reserve (international)
Mozambique MOZ	Estuary 473	Nova Mambone, Inhambane province	Gross Gain %	Remote area allows regeneration from natural hydrodynamic processes.	No
	Estuary 493				
	Estuary 927	Mogincual Nampula Province			
Venezuela, Bolivian Republic of VEN	Open Coast 61757	Near border with Guyana	Gross Gain %	Large, protected area allows regeneration from natural hydrodynamic processes.	Imataca Forest Reserve (national)
**LOW GAIN**					
Belize BLZ	Lagoon 20113	Corozal, north of Belize City	Gross Gain %	Adjoins protected marine reserve – possible hydrology changes from highway construction (Old Northern Hwy).	Partially – Hol Chan marine reserve and Corozal Bay wildlife sanctuary (national)

Supplementary Table 3. Summary of response variables. Number of observations for each variable (n) given removal of mangrove geomorphic units less than 1 ha to reduce mapping uncertainty.

Variable	Rationale	Description	Value	Data Source	Scale	n	
						1996-2007	2007-2016
Net Change	An indicator of conservation failure or threat (loss) and conservation or restoration success (gain or no change) over time.	Net area of mangrove cover increase or decrease between 1996-2007 and 2007-2016 per geomorphic unit.	km^2 (-ve and +ve)	Global Mangrove Watch v2.0[1] (no data available for 2006)	Landscape Mangrove typology v2.2[2]	4235	4235
Net Loss [log]	An indicator of conservation failure or threat over time.	Net area of mangrove cover decrease between 1996-2007 and 2007-2016 per geomorphic unit.	km^2 (+ve)			2880	2706
% Net Loss [log]	An indicator of conservation failure or threat over time.	Net area of mangrove cover decrease between 1996-2007 and 2007-2016 per typological unit, divided by the initial area of mangrove cover.	% (+ve)			2880	2706
Net Gain [log]	An indicator of conservation or restoration success over time.	Net area of mangrove cover increase between 1996-2007, and 2007-2016 per typological unit.	km^2 (+ve)			728	1138
% Net Gain [log]	An indicator of conservation or restoration success over time.	Net area of mangrove cover increase between 1996-2007, and 2007-2016 per typological unit, divided by the initial area of mangrove cover.	% (+ve)			728	1138
Gross Loss [log]	An indicator of conservation failure or threat over time, not offset by any gains in the landscape.	Absolute area of mangrove cover decrease between 1996-2007, and 2007-2016 per typological unit (not accounting for any increases in area).	km^2 (+ve)			3512	3718
% Gross Loss [log]	An indicator of conservation failure or threat over time, not	Absolute area of mangrove cover decrease between 1996-2007, and 2007-2016 per	% (+ve)			3512	3718

Variable	Rationale	Description	Value	Data Source	Scale	n	
						1996-2007	2007-2016
	offset by any gains in the landscape.	typological unit, divided by the initial area of mangrove cover (not accounting for any increases in area).					
Gross Gain [log]	An indicator of conservation or restoration success over time, not offset by any losses in the landscape.	Absolute area of mangrove cover increase between 1996-2007, and 2007-2016 per typological unit (not accounting for any decreases in area).	km^2 (+ve)			3182	3479
% Gross Gain [log]	An indicator of conservation or restoration success over time, not offset by any losses in the landscape.	Absolute area of mangrove cover increase between 1996-2007, and 2007-2016 per typological unit, divided by the initial area of mangrove cover (not accounting for any decreases in area).	% (+ve)			3182	3479

Supplementary Table 4. Summary of socio-economic variables. Number of observations for each variable (n) given removal of geomorphic units less than 1 ha.

Driver	Rationale	Variable	Description	Value	Data source	Scale	n units	n countries
Economic growth	Whilst a country's wealth was found to be associated with mangrove loss[3], it can also improve governance and decrease environmental pressure[4]. Night-time lights are local measures of economic output that trend closely with economic growth particularly in coastal regions[5,6].	Night-time lights growth	Change in annual average stable lights within 100 km buffer of centroid of mangrove typology unit from 1996-2007, and 2007-2013 (no data available past 2013). Stable lights data are available at 30 arc-second resolution (approximately 1 km^2 at the equator), and contains the annual average visible band light intensity, reported as a digital number ranging from 0-63.	Digital number	Defense Meteorological Satellite Program – Operational Linescan System (DMSP-OLS) Night-time Lights Time Series v.4, 1992-2013, Stable Lights[7].	Landscape	4235	108
Market accessibility	Travel time to the nearest major market (capital, city or major population centre), and combination of the size (population) and travel time to the nearest major market,	Travel time to nearest city	Mean travel time from typological unit to nearest city in 2015 via surface transport. The global accessibility map shows the travel	Minutes	Global map of travel time to cities in 2015[11,12]	Landscape	4051	108

Driver	Rationale	Variable	Description	Value	Data source	Scale	n units	n countries
	termed "market gravity" have been shown to be strong predictors of fish biomass on coral reefs[8-10]. Access to markets to trade commodities (e.g. rice, shrimp and oil palm) is also likely to drive mangrove loss from conversion to aquaculture and agriculture.		time in minutes to high-density urban centres for 2015 at a 30 arc-second resolution (1 km^2 raster pixels at the equator). It uses the Global Human Settlement Grid of high-density land cover to represent cities and a global friction surface of roads and land cover to quantify the travel time of moving through a least-cost-path between each pixel and its nearest high-density urban point[7].					
Economic complexity	Corruption distorts environmental regulations, facilitating deforestation and promoting poverty and illegal activities[13]. Decreases in environmental pressures on terrestrial biodiversity	Economic Complexity Index (ECI)	The ECI measures a country's economic capacity inferred from data connecting locations (e.g. countries, cities, regions) to the activities that are present in them (e.g. products,	Index -2.5 to +2.5	OEC Economic Complexity Index 1995 – 2018 HS92 4-digit[14]	National	3594 (1996); 3793 (2007); 3843 (2016)	50 (1996); 61 (2007); 65 (2016)

Driver	Rationale	Variable	Description	Value	Data source	Scale	n units	n countries
	have been discovered in the wealthiest countries and those with strong control of corruption[4].		industries, technologies). Adopted 2007 and 2016 indices as greater coverage of countries.					
Democracy	Protected areas were more effective in avoiding deforestation in countries with higher levels of control of corruption and democracy[15]. Democracy has also been found to influence Nationally Determined Contribution (NDC) ambition[16].	Varieties of Democracy (VDEM)	Country scores on the VDEM multiplicative polyarchy index measures a country's degree of freedom of association, clean elections, freedom of expression, elected executives and suffrage. Adopted 2007 and 2016 indices to maintain consistency with adoption of ECI indices.	Index 0-1	Varieties of Democracy (V-Dem) dataset v.10[17]	National	4035 (1996, 2007 and 2016)	80
Environmental governance	Improvements in environmental governance can help protect marine ecosystems[18].	Environmental Performance Index (EPI) Biodiversity and Habitat (BDH) issue category	The EPI BDH scores countries based on: Terrestrial Biome Protection (national) (20%), Terrestrial Biome Protection (global) (20%), Marine	Index 0-100	Environmental Performance Index 2020[19]	National	4116	89

Driver	Rationale	Variable	Description	Value	Data source	Scale	n units	n countries
			Protected Areas (20%), Protected Areas Representativeness Index (10%), Species Habitat Index (10%), Species Protection Index (10%), and Biodiversity Habitat Index (10%).					
Community forestry	In south-east Asia and east Africa, community forestry programs are being encouraged in mangrove areas that are under pressure from resource harvesting[20,21]. Community forestry has been found to reduce deforestation and poverty in terrestrial forests in Kalimantan, Indonesia however its effects varied across social and biophysical contexts[22]. Despite the promotion of community forestry in developing	Community forestry effort	Each country was scored 0-3 based on the following criteria: +1 (1-50 community forestry case studies); +2 (>50 community forestry case studies – the threshold was based on the 90th percentile of the data range and indicates that community forestry was widespread; +1 (community forestry case study/ies existed in mangroves in particular).	Index 0-3	Review of published literature for mangrove-holding countries to identify how many community forestry case studies were reported in each country, and whether any were in mangroves.	National	4235	109

Driver	Rationale	Variable	Description	Value	Data source	Scale	n units	n countries
	countries to improve livelihoods and conservation[23], the benefits of community forestry on mangrove conservation have not been assessed.							
Indigenous land	Indigenous land tenure can promote more successful community forestry[24] and has been found to reduce deforestation rates globally[25] and improve the success of community-based mangrove restoration projects[26,27].	Indigenous land tenure	The proportion of Indigenous peoples' land verse other land per country.	Proportion	Global Indigenous land[28]. Only country-level data published. There is a global map in the article, but no available spatial data.	National	4124	106
Climate commitment	Many countries are including coastal wetlands in their national climate policies, known as NDCs in response to the Paris Agreement[29].<br>It is likely that a country's commitment to include mangroves or coastal ecosystems in their actions to	Nationally Determined Contributions (NDC) commitments	Each country was scored 0-4 based on the following criteria:<br>+1 (NDC submitted, first or second);<br>+1 (NDC uses the IPCC Wetlands Supplement (IPCC 2013) or mentions mangroves or coastal ecosystems in accounting);	Index 0-4	Review of First NDCs in the NDC register	National	4211	103

Driver	Rationale	Variable	Description	Value	Data source	Scale	n units	n countries
	mitigate and/or adapt to climate change indicates a better baseline for mangrove conservation.		+1 (NDC mentions mangroves or coastal ecosystems in mitigation); +1 (NDC mentions mangroves or coastal ecosystems in adaptation).					
Restoration	The number of mangrove restoration projects has been growing steadily since the 1970s and large-scale restoration will be critical to recover the loss and degradation of mangroves[30].	Mangrove restoration effort	Number of mangrove restoration projects per country reported in published and grey literature.	Frequency	Mangrove restoration databases (Lovelock unpublished, Gott unpublished)	National	4235	76 (33 countries with no restoration detected)
Protected area management	Protected area coverage has been found to have a positive effect on mangrove cover at the global scale[31]. Other global studies of the socioeconomic factors that favour conservation of marine ecosystems have found strong effects of governance of marine protected areas (MPAs),	Marine Protected Area (MPA) staff capacity	Review of the staff capacity of 433 MPAs globally, based on the Management Effectiveness Tracking Tool (METT), the World Bank MPA Score Card, and the NOAA Coral Reef Conservation Program's (CRCP) MPA Management	Index 1-3	MPA management data[32].	National	3084	57

Driver	Rationale	Variable	Description	Value	Data source	Scale	n units	n countries
	including adequate staff and budget capacity[32] and high compliance in protected areas[8]. Adequate staff capacity was the most important factor in explaining fish responses to MPA protection, with budget capacity the second, but they were significantly correlated[32].		Assessment Checklist.					
Wetland conservation	The Ramsar Convention on Wetlands provides the framework for the conservation and wise use of wetlands. The ecological character of Ramsar listed wetlands have been found to be significantly better than those of wetlands generally[33].	Ramsar wetlands	Area of Ramsar listed coastal and marine wetlands per country	Ha	Ramsar Sites Information Service (https://rsis.ramsar.org/)	National	4235	71 ((38 countries with no Ramsar wetlands)

Supplementary Table 5. Biophysical variables. Number of observations for each variable (n) after removal of geomorphic units less than 1 ha.

Driver	Rationale	Variable	Description	Value	Data source	Scale	n units
Geomorphology	Geomorphic setting influences the value and loss rate of mangroves. Although decline in area has been shown in all types (Deltas, Estuaries, Lagoons, Open Coast), the losses of lagoonal mangroves were nearly twice those of other types[2]. Furthermore, the stronger incorporation of geomorphic knowledge into site planning and design of mangrove restoration will improve success rates[34].	Mangrove typology	Classification of mangrove typologies into Deltas, Estuaries, Lagoons and Open Coast.	Categorical	Mangrove Typology v2.2[2]	Landscape	4235
Sediment availability	Mangrove retreat and expansion are driven by sediment deposit and erosion, which are in turn influenced by sediment	Sediment trapping index	Weighted-mean sediment trapping index of rivers within typological units. Sediment trapping index based on connectivity status	Percentage	Global free-flowing river dataset[39]	Landscape	4235

Driver	Rationale	Variable	Description	Value	Data source	Scale	n units
	availability from rivers and wave action, and alterations in hydrological regimes[35-37]. Sediment retention because of dams in rivers has been found to contribute significantly to mangrove decline on a global-scale[38].		index (CSI) of river reaches assessed on: (1) longitudinal (connectivity between up- and downstream), (2) lateral (connectivity to floodplain and riparian areas), (3) vertical (connectivity to groundwater and atmosphere), and (4) temporal (connectivity based on seasonality of flows). Lower sediment trapping index is associated with higher levels of connectivity throughout the river reach, and therefore higher sediment availability from rivers.				
Habitat fragmentation	There is not a clear relationship between habitat fragmentation and mangrove loss globally (e.g. high fragmentation in areas with low rates of loss). However, habitat fragmentation has been correlated to loss and	Clumpiness	Mangrove patch clumpiness at the start of each time period (1996 and 2007) calculated using the Global Mangrove Watch dataset for 2016 following the methods in Bryan-Brown, et al. [40].	Index	Global Mangrove Watch dataset for 2016 v.2.0[1]	Landscape	4231 (1996); 4233 (2007)

Driver	Rationale	Variable	Description	Value	Data source	Scale	n units
	degradation rates in several countries[40].						
Tidal amplitude	Tidal range and hydroperiod (which in turn affects nutrient availability and soil salinity) are important for mangrove structure, height, and productivity at local and regional scales[41]. High tidal amplitudes, temperature and rainfall have been found to increase mangrove biomass at continental scales[42]. Tidal amplitude could also be related to mangrove loss if there are larger tidal flat areas for aquaculture or agriculture.	Tidal amplitude	FES2014 integrates altimeter data from multiple satellites into a 2/3-D ocean hydrodynamics model[43]. Tidal amplitude raster with a pixel resolution of 1/16° (~7km at the equator).<br>Mean tidal amplitude for each typological unit quantified based on the mean-weighted average of proximate pixel values of mangrove patches within each unit.	Continuous	Finite Element Solution global tide model (FES2014)[44] https://www.aviso.altimetry.fr	Landscape	4235
Antecedent sea-level rise	The distribution of mangroves on shorelines changes over time with sediment	Antecedent sea level rise	The weighted-mean antecedent sea-level rise per geomorphic unit was estimated from the regional mean sea-	Continuous	European Space Agency (ESA) Climate Change Initiative (CCI)<br>global sea level ECV product v.2.0[50]	Landscape	4235

Driver	Rationale	Variable	Description	Value	Data source	Scale	n units
	accretion, erosion, subsidence, and sea-level rise[45,46]. Mangroves are likely to have persisted in regions with long periods of sea-level stability, and whilst millennial-scale sea-level rise may have led to mangrove expansion, depending on the available accommodation space[47], sea-level variability and periods of low sea-level can cause mangrove dieback[48,49].		level trends dataset between January 1993 and December 2015 (raster pixel resolution of 1/4°, ~28km at the equator).		(http://www.esa-sealevel-cci.org/)		
Drought	Whilst long-term precipitation and temperature influence mangrove distribution globally[51], periods of low rainfall have been reported to cause extensive mangrove dieback	Standardized Precipitation-Evapotranspiration Index (SPEI)	SPEI expresses the deviations of the current climatic balance (precipitation minus evapotranspiration potential) with respect to the long-term balance. The global SPEI database provides SPEI values at a 0.5° spatial resolution and a	Index (scale + to -)	Global SPEI database (SPEIbase) v.2.6 since 1900[53-55]	Landscape	4073

Driver	Rationale	Variable	Description	Value	Data source	Scale	n units
	at regional scales, particularly combined with high temperatures and a drop in sea-level[47,52].		monthly time resolution. The mean SPEI per geomorphic unit was calculated for each time series (1996-2006, 2007-2016).				
Tropical storms	Large-scale destruction of mangroves across regions have been reported from tropical storms inducing strong winds, high energy waves and storm surges[56,57].	Tropical storm frequency	Tropical storm frequency was calculated as the number of tropical storm occurrences (points along their paths) within a 200 km buffer of the centroid of geomorphic units within each time series (1996-2006, and 2007-2016). IBTrACS provides location and intensity for global tropical storms (cyclones and hurricanes) since the 1840s, generally providing data at 3-hour intervals.	Count	International Best Track Archive for Climate Stewardship (IBTrACS) v.4 dataset 1980-2020[58]	Landscape	4235
Extreme low temperature	Extreme low temperature events were a driver of mangrove loss in subtropical regions, such as Florida and	Minimum temperature of the coldest month	WorldClim bioclimatic variable 6 (BIO6) was used to calculate the average minimum temperature (°C) across mangrove geomorphic units. BIO6 is the	Continuous	WorldClim bioclimatc variables 2.5 minutes[63,64]	Landscape	4235

Driver	Rationale	Variable	Description	Value	Data source	Scale	n units
	Louisianan of the US and China [59-61]. The intensity of extreme low temperature would be increased with future climate change, impacting mangroves located in the northern or southern limits[62].		average minimum temperature for the years 1970-2000 at a spatial resolution of 2.5 minutes (~21 km^2).				

Supplementary Table 6. Correlation matrix of 17 potential socioeconomic and biophysical explanatory variables of mangrove change for 1996-2007 showing Pearson's correlation coefficient (*r*) for full dataset. VDEM = Varieties of Democracy, ECI = Environmental Complexity Index, EPI BDH = Environmental Performance Index Biodiversity and Habitat Category, MPA = Marine protected area, NDC = Nationally Determined Contribution, CF = Community forestry, SLR = sea-level rise, SPEI = Standardized Precipitation Evapotranspiration Index, Tmin = minimum temperature of the coldest month. Combinations with $r > 0.5$ are shown in bold. Number of observations (*n*) for each variable is shown on the diagonal given the removal of mangrove geomorphic units less than 1 ha.

	Night lights 96-07	Travel time	VDEM 1996	ECI 2007	EPI BDH 2010	MPA staff	NDC commitment	CF effort	Restoration effort	Indigenous land	Ramsar wetland	Tidal amplitude	Historic SLR	Sediment trapping	Tropical storms 96-06	SPEI 96-06	Clumpiness 1996	Tmin
Nightlights 96-07	*4235*																	
Travel time	-0.23	*4051*																
VDEM 1996	-0.10	0.18	*4035*															
ECI 2007	0.24	-0.22	0.20	*3793*														
EPI BDH 2010	-0.15	-0.08	0.32	0.34	*4116*													
MPA staff	0.17	-0.15	-0.33	**0.54**	0.32	*3084*												
NDC commitment	-0.03	0.13	-0.04	-0.17	-0.11	0.04	*4211*											
CF effort	-0.10	-0.06	-0.28	0.13	0.08	0.40	0.24	*4235*										
Restoration effort	0.09	-0.16	-0.20	0.35	0.11	0.34	-0.49	0.36	*4235*									
Indigenous land	-0.01	0.04	-0.01	0.18	0.31	0.43	0.31	0.42	0.11	*4124*								
Ramsar wetland	0.00	-0.02	-0.03	-0.08	-0.02	0.05	-0.01	-0.04	-0.04	0.02	*4235*							
Tidal amp.	-0.01	0.13	0.27	-0.06	0.08	-0.03	0.17	0.02	-0.14	0.16	-0.03	*4235*						
Historic SLR	-0.10	0.10	-0.15	-0.14	-0.05	-0.08	-0.01	0.17	0.24	0.20	-0.02	-0.07	*4235*					

	Night lights 96-07	Travel time	VDEM 1996	ECI 2007	EPI BDH 2010	MPA staff	NDC commitment	CF effort	Restoration effort	Indigenous land	Ramsar wetland	Tidal amplitude	Historic SLR	Sediment trapping	Tropical storms 96-06	SPEI 96-06	Clumpiness 1996	Tmin
Sediment trapping	0.08	-0.11	0.06	0.09	-0.02	0.03	-0.03	-0.02	-0.03	-0.06	0.08	-0.02	-0.11	*4235*				
Tropical storms 96-06	0.10	0.15	0.26	0.19	-0.04	0.13	-0.18	-0.18	0.30	-0.02	-0.02	0.12	0.03	-0.01	*4235*			
SPEI 96-06	-0.11	0.19	0.23	-0.03	0.10	-0.02	-0.12	0.05	0.16	0.09	-0.09	0.17	0.16	-0.04	0.31	*4073*		
Clumpiness 1996	0.00	0.00	0.05	0.00	0.00	-0.09	-0.01	-0.08	-0.04	-0.05	0.00	-0.02	-0.01	-0.01	0.02	0.00	*4231*	
Tmin	-0.18	0.05	-0.42	-0.22	0.02	-0.12	-0.13	0.19	0.21	0.07	-0.03	-0.18	0.27	-0.11	-0.22	0.18	-0.03	*4235*

Supplementary Table 7. Correlation matrix of 17 potential socioeconomic and biophysical explanatory variables of mangrove change for 2007-2016 showing Pearson's correlation coefficient (*r*) for full dataset. VDEM = Varieties of Democracy, ECI = Environmental Complexity Index, EPI BDH = Environmental Performance Index Biodiversity and Habitat Category, MPA = Marine protected area, NDC = Nationally Determined Contribution, CF = Community forestry, SLR = sea-level rise, SPEI = Standardized Precipitation Evapotranspiration Index, Tmin = minimum temperature of the coldest month. Combinations with $r > 0.5$ are shown in bold. Number of observations (*n*) for each variable is shown on the diagonal given the removal of mangrove geomorphic units less than 1 ha.

	Night lights 07-13	Travel time	VDEM 2007	ECI 2016	EPI BDH 2020	MPA staff	NDC commitment	CF effort	Restoration effort	Indigenous land	Ramsar wetland	Tidal amplitude	Antecedent SLR	Sediment trapping	Tropical storms 07-16	SPEI 07-16	Clumpiness 2007	Tmin
Nightlights 07-13	*4235*																	
Travel time	-0.27	*4051*																
VDEM 2007	-0.22	0.21	*4035*															
ECI 2016	0.27	-0.28	0.09	*3843*														
EPI BDH 2020	-0.10	0.02	**0.60**	0.16	*4116*													

	Night lights 07-13	Travel time	VDEM 2007	ECI 2016	EPI BDH 2020	MPA staff	NDC commitment	CF effort	Restoration effort	Indigenous land	Ramsar wetland	Tidal amplitude	Antecedent SLR	Sediment trapping	Tropical storms 07-16	SPEI 07-16	Clumpiness 2007	Tmin
MPA staff	0.10	-0.15	0.18	**0.55**	0.28	*3084*												
NDC commitment	-0.03	0.13	0.26	-0.28	0.03	0.04	*4211*											
CF effort	-0.03	-0.06	0.30	0.18	-0.06	0.40	0.24	*4235*										
Restoration effort	0.05	-0.16	-0.07	0.50	-0.08	0.34	-0.49	0.36	*4235*									
Indigenous land	0.01	0.04	0.29	0.15	0.24	0.43	0.31	0.42	0.11	*4124*								
Ramsar wetland	-0.01	-0.02	-0.05	-0.06	-0.04	0.05	-0.01	-0.04	-0.04	0.02	*4235*							
Tidal amplitude	0.02	0.13	0.19	-0.11	0.14	-0.03	0.17	0.02	-0.14	0.16	-0.03	*4235*						
Antecedent SLR	-0.10	0.10	-0.01	-0.10	-0.20	-0.08	-0.01	0.17	0.24	0.20	-0.02	-0.07	*4235*					
Sediment trapping	0.12	-0.11	0.00	0.06	0.02	0.03	-0.03	-0.02	-0.03	-0.06	0.08	-0.02	-0.11	*4235*				
Tropical storms 07-16	-0.06	0.13	0.04	0.14	0.13	0.16	-0.14	-0.19	0.23	-0.02	-0.02	0.12	0.03	-0.01	*4235*			
SPEI 07-16	-0.08	0.08	0.00	0.12	-0.11	0.11	-0.12	0.15	0.36	0.18	-0.09	-0.01	0.18	-0.04	0.12	*4073*		
Clumpiness 2007	-0.01	0.01	-0.03	-0.04	-0.03	-0.12	-0.03	-0.08	-0.06	-0.05	0.00	-0.01	-0.01	-0.01	-0.01	-0.03	*4233*	
Tmin	-0.12	0.05	-0.22	-0.10	-0.25	-0.12	-0.13	0.19	0.21	0.07	-0.03	-0.18	0.27	-0.11	-0.25	0.33	-0.02	*4235*

Supplementary Table 8. Models fitted for each of nine response variable – Net Change, Net Loss, % Net Loss, Net Gain, % Net Gain, Gross Loss, % Gross Loss, Gross Gain, % Gross Gain. SLR = sea-level rise, NDC = Nationally Determined Contribution, tmin = minimum temperature of the coldest month. # geomorphic unit size only included for area response variables.

Model	Time series	Level 1 predictors (individual)	Level 2 predictors (country)	Random intercept	Random slopes
Null linear<br>$y_i = \beta_0 + e_i$	1996-2007<br>2007-2016	NA	NA	NA	NA
Null random intercept<br>$y_{ij} = \beta_0 + u_j + e_{ij}$	1996-2007<br>2007-2016	NA	NA	Country	NA
Random intercept<br>$y_{ij} = \beta_0 + \beta_1 X_{1ij} + \beta_2 X_{2ij} + \beta_3 X_{3ij} + \ldots + u_j + e_{ij}$	1996-2007	Night-time lights growth 1996-2007, travel time to nearest city, tropical storm frequency 1996-2006, drought 1996-2006, tidal amplitude, antecedent SLR, sediment trapping, clumpiness 1996, tmin, mangrove typology, geomorphic unit size#	Democracy 1996, economic complexity 2007, NDC commitment, community forestry effort, Indigenous land, restoration effort	Country	NA
Random coefficients (random intercept and slopes)	1996-2007	Night-time lights growth 1996-2007, travel time to nearest city, tropical storm frequency 1996-2006, drought 1996-2006, tidal amplitude, antecedent SLR, sediment trapping, clumpiness 1996, tmin, mangrove typology, geomorphic unit size#	Democracy 1996, economic complexity 2007, NDC commitment, community forestry effort, Indigenous land, restoration effort	Country	Night-time lights growth 1996-2007, travel time to nearest city
Spatial autoregressive (neighbourhood distance 500km)	1996-2007	Night-time lights growth 1996-2007, travel time to nearest city, tropical storm frequency 1996-2006, drought 1996-2006, tidal amplitude, antecedent SLR, sediment trapping, clumpiness 1996, tmin, mangrove typology, geomorphic unit size#	Democracy 1996, economic complexity 2007, NDC commitment, community forestry effort, Indigenous land, restoration effort	NA	NA
Random intercept	2007-2016	Night-time lights growth 2007-2013, travel time to nearest city, tropical storm frequency 2007-2016, drought 2007-2016, tidal amplitude, antecedent SLR, sediment trapping, clumpiness 2007, tmin, mangrove typology, geomorphic unit size#	Democracy 2007, economic complexity 2016, NDC commitment, community forestry effort, Indigenous land, restoration effort	Country	NA
Random coefficients (random intercept and slopes)	2007-2016	Night-time lights growth 2007-2013, travel time to nearest city, tropical storm frequency 2007-2016, drought 2007-2016, tidal amplitude, antecedent SLR, sediment trapping, clumpiness 2007, tmin, mangrove typology, geomorphic unit size#	Democracy 2007, economic complexity 2016, NDC commitment, community forestry effort, Indigenous land, restoration effort	Country	Night-time lights growth 2007-2013, travel time to nearest city
Spatial autoregressive (neighbourhood distance 500km)	2007-2016	Night-time lights growth 2007-2013, travel time to nearest city, tropical storm frequency 2007-2016, drought 2007-2016, tidal amplitude, antecedent SLR, sediment trapping, clumpiness 2007, tmin, mangrove typology, geomorphic unit size#	Democracy 2007, economic complexity 2016, NDC commitment, community forestry effort, Indigenous land, restoration effort	NA	NA

Supplementary Table 9. Likelihood ratio test between (a) null linear model and random intercept null model and (b) random intercept model and random coefficients model. If p-value is significant (<0.05), there is justification to include (a) country as random intercept and (b) Night-time lights growth and travel time to nearest city as random slopes.

Response variable	a) Effect of country as random intercept	b) Effect of night-time lights and travel time as random slopes	Model adopted (AIC, df and R^2m)	Residuals plot
Net Change 1996-2007 km^2	8.11 on 1 d.f. p=0.004	52.08 on 5 d.f. p<0.0001	Random coefficients (23321, 28 d.f., 0.24)<br>Uneven residuals, not adopted.	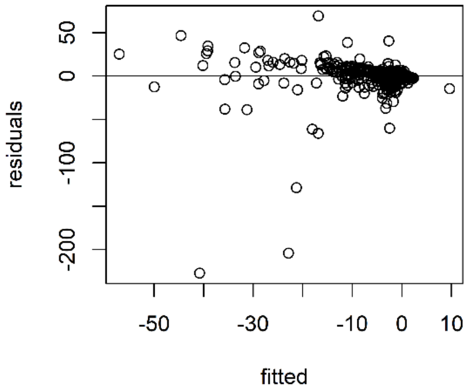
Net Change 2007-2016 km^2	18.61 on 1 d.f. p<0.0001	14.13 on 5 d.f. p=0.01	Random coefficients (19456, 28 d.f., 0.18)<br>Uneven residuals, not adopted.	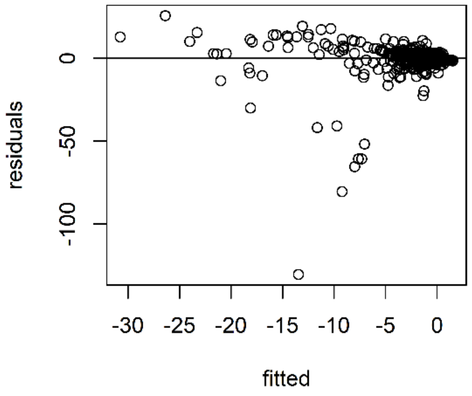

Response variable	a) Effect of country as random intercept	b) Effect of night-time lights and travel time as random slopes	Model adopted (AIC, df and R^2m)	Residuals plot
% Net Change 1996-2007	10.82 on 1 d.f. p=0.001	2.46 on 5 d.f., p=0.78	Random intercept (27507, 22 d.f., 0.03)<br>Uneven residuals, not adopted.	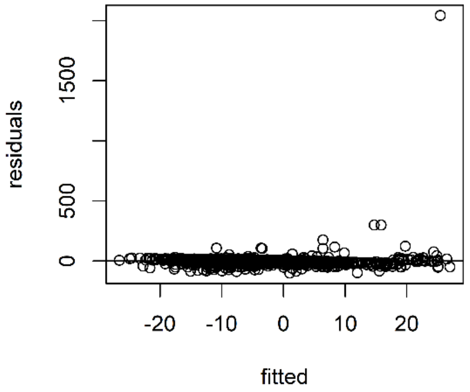
% Net Change 2007-2016	0.06 on 1 d.f.<br>p=0.80	NA	Linear model (26202, 21 d.f., 0.006)<br>Uneven residuals, not adopted.	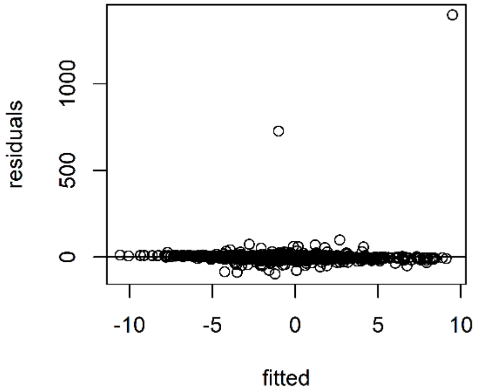

Response variable	a) Effect of country as random intercept	b) Effect of night-time lights and travel time as random slopes	Model adopted (AIC, df and R^2m)	Residuals plot
Log (Net Loss 1996-2007 km^2)	204.69 on 1 d.f. p<0.001	48.18 on 5 d.f. p<0.001	Random coefficient (9941 d.f., 0.29)<br>Uneven residuals, not adopted.	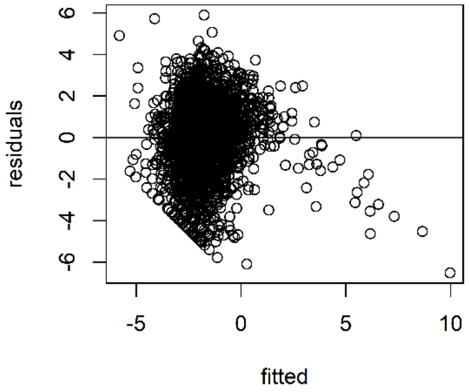
Log (Net Loss 2007-2016 km^2)	53.62 on 1 d.f. p<0.001	31.34 on 5 d.f. p<0.001	Random coefficient (9250, 28 d.f., 0.26)<br>Uneven residuals, not adopted.	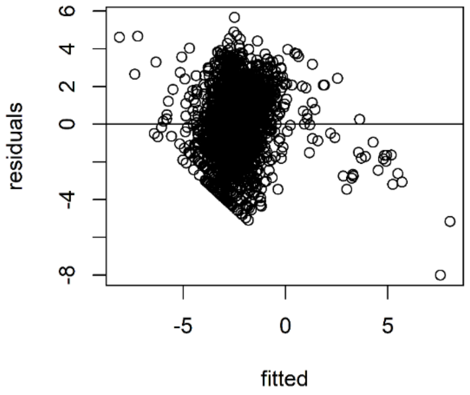

Response variable	a) Effect of country as random intercept	b) Effect of night-time lights and travel time as random slopes	Model adopted (AIC, df and R^2m)	Residuals plot
Log (% Net Loss 1996-2007)	286.62 on 1 d.f. p<0.001	70.15 on 5 d.f. p<0.001	Random coefficient (6787, 27 d.f., 0.15)	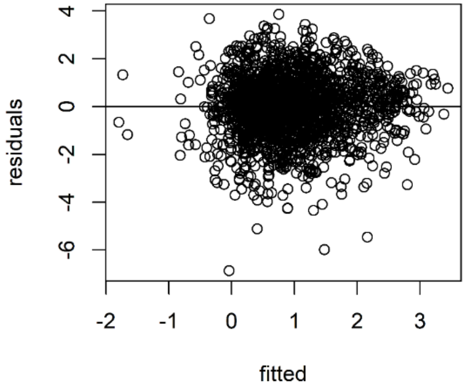
Log (% Net Loss 2007-2016)	67.24 on 1 d.f. p<0.001	38.36 on 5 d.f. p<0.001	Random coefficient (6728, 27 d.f., 0.07)	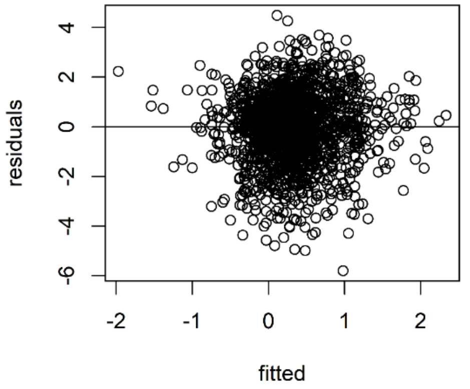

**Response variable**	**a) Effect of country as random intercept**	**b) Effect of night-time lights and travel time as random slopes**	**Model adopted (AIC, df and R^2m)**	**Residuals plot**
Log (Gross Loss 1996-2007 km^2)	211.44 on 1 d.f. p<0.001	54.43 on 5 d.f. p<0.001	Random coefficient (12013, 28 d.f., 0.29)	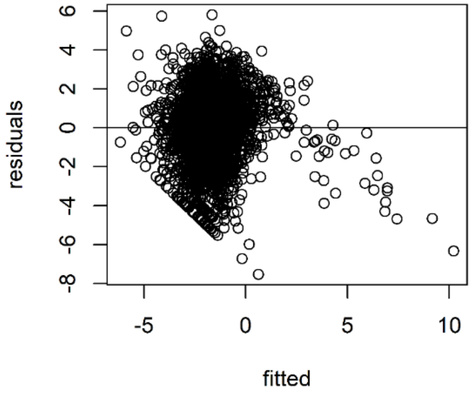
Log (Gross Loss 2007-2016 km^2)	128.99 on 1 d.f. p<0.001	43.27 on 5 d.f. p<0.001	Random coefficient (12531, 28 d.f., 0.32)	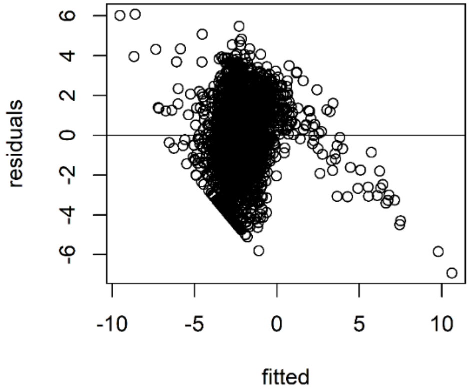

Response variable	a) Effect of country as random intercept	b) Effect of night-time lights and travel time as random slopes	Model adopted (AIC, df and R^2m)	Residuals plot
Log (% Gross Loss 1996-2007)	357.70 on 1 d.f. p<0.001	103.46 on 5 d.f. p<0.001	Random coefficient (7883, 27 d.f., 0.16)	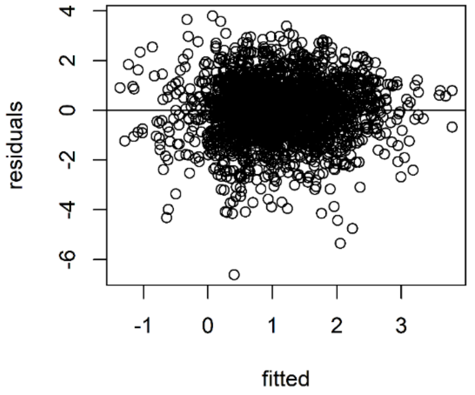
Log (% Gross Loss 2007-2016)	236.64 on 1 d.f. p<0.001	79.67 on 5 d.f. p<0.001	Random coefficient (8104, 27 d.f., 0.08)	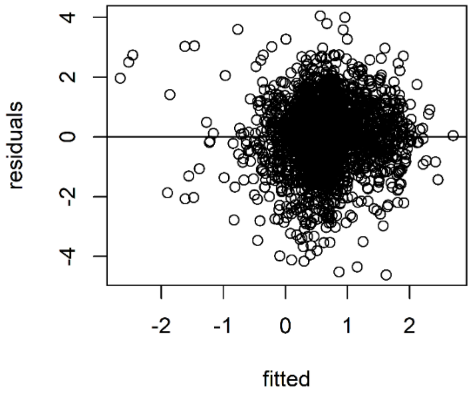

Response variable	a) Effect of country as random intercept	b) Effect of night-time lights and travel time as random slopes	Model adopted (AIC, df and R^2m)	Residuals plot
Log (Net Gain 1996-2007 km^2)	99.93 on 1 d.f. p<0.001	15.15 on 5 d.f. p=0.009	Random coefficients (2326, 28 d.f., 0.43)	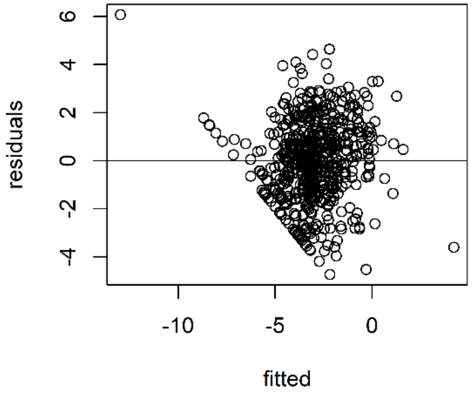
Log (Net Gain 2007-2016 km^2)	74.02 on 1 d.f. p<0.001	28.18 on 5 d.f. p<0.001	Random coefficients (3724, 28 d.f. 0.37)	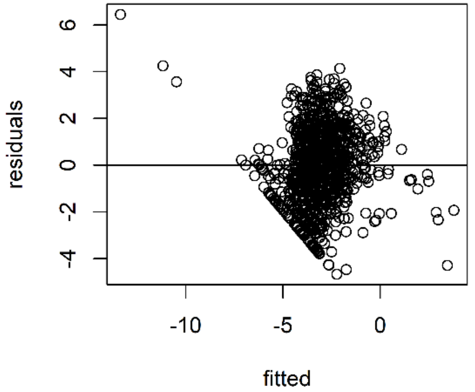

Response variable	a) Effect of country as random intercept	b) Effect of night-time lights and travel time as random slopes	Model adopted (AIC, df and R^2m)	Residuals plot
Log (% Net Gain 1996-2007)	154.12 on 1 d.f. p<0.001	13.00 on 5 d.f. p=0.02	Random coefficient (1727, 27 d.f., 0.40)	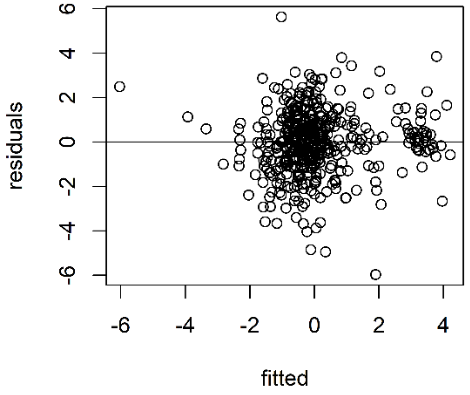
Log (% Net Gain 2007-2016)	35.22 on 1 d.f. p<0.001	23.84 on 5 d.f., p<0.001	Random coefficient (2756, 27 d.f., 0.14)	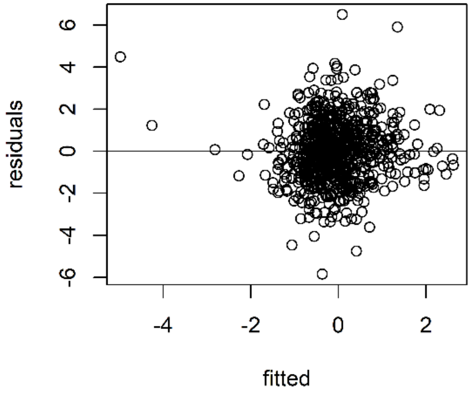

Response variable	a) Effect of country as random intercept	b) Effect of night-time lights and travel time as random slopes	Model adopted (AIC, df and R^2m)	Residuals plot
Log (Gross Gain 1996-2007 km^2)	209.42 on 1 d.f. p<0.001	25.06 on 5 d.f. p<0.001	Random coefficients (10310, 28 d.f., 0.41)	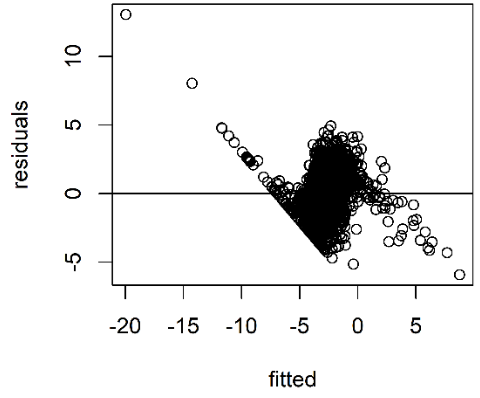
Log (Gross Gain 2007-2016 km^2)	229.86 on 1 d.f. p<0.001	0 on 5 d.f.<br>p=1	Random intercept (9978, 23 d.f., 0.15)	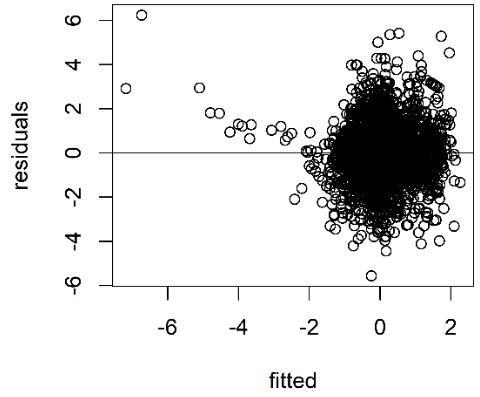

**Response variable**	**a) Effect of country as random intercept**	**b) Effect of night-time lights and travel time as random slopes**	**Model adopted (AIC, df and R^2m)**	**Residuals plot**
Log (% Gross Gain 1996-2007)	276.42 on 1 d.f. p<0.001	60.63 on 5 d.f., p<0.001	Random coefficients (7696, 27 d.f., 0.26)	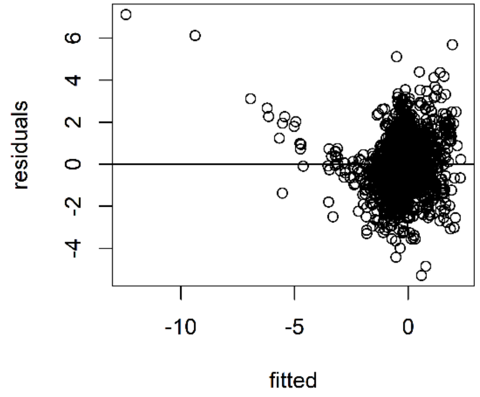
Log (% Gross Gain 2007-2016)	461.12 on 1 d.f., p<0.001	86.61 on 5 d.f. p<0.001	Random coefficients (8112, 27 d.f., 0.15)	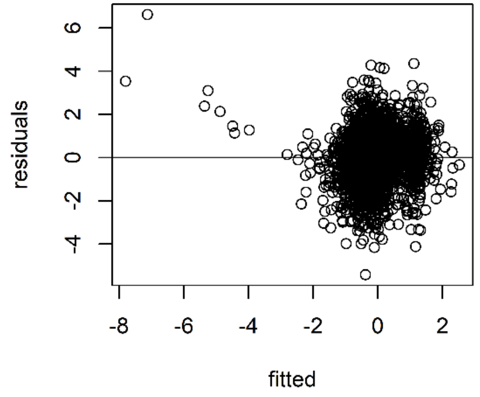

Supplementary Table 10. Significant effects of socioeconomic (SE) and biophysical (BP) variables (estimates, confidence intervals [*CI*], *t*-values, and *p*-values) across mangrove cover change variables and time periods. NDC = Nationally Determined Contribution, SLR = sea-level rise, Tmin = minimum temperature. Asterisks indicate significant effects at p<0.05*, p<0.01**, and p<0.001***. *ICC* = Intraclass correlation coefficient, R^2m/c = marginal/conditional R^2, *df* = degrees of freedom, *n* = sample size.

Response		1996-2007					2007-2016				
		**Variable**	**est.**	***CI***	***t***	***p***	**Variable**	**est.**	***CI***	***t***	***p***
Net Loss %	SE	Restoration effort	0.27	0.08 – 0.45	2.83	0.005*	Restoration effort	0.18	0.06 – 0.29	3.05	0.002**
		Travel time to nearest city	-0.14	-0.26 – -0.02	-2.21	0.027*	Travel time to nearest city	-0.16	-0.25 – -0.08	-3.66	<0.001***
	BP	Clumpiness	-0.01	-0.02 – -0.00	-2.91	0.004**	Clumpiness	-0.03	-0.04 – -0.01	-3.52	<0.001***
		Tidal amplitude	-0.20	-0.28 – -0.13	-5.17	<0.001***	Tidal amplitude	-0.12	-0.20 – -0.05	-3.16	0.002**
		Antecedent SLR	0.08	0.02 – 0.14	2.68	0.007**					
		Drought	-0.28	-0.48 – -0.09	-2.80	0.005**					
		Tmin	-0.05	-0.07 – -0.02	-3.21	0.001**					
		Delta	2.42	1.25 – 3.59	4.05	<0.001***					
		Estuary	2.37	1.25 – 3.50	4.12	<0.001***					
		Lagoon	2.36	1.23 – 3.50	4.09	<0.001***					
		Open Coast	2.38	1.25 – 3.51	4.13	<0.001***					
		ICC = NA, R^2m/c = 0.17/NA, *df* = 27, *n* = 2004, countries = 53					*ICC* = 0.06, R^2m/c = 0.07/0.13, *df* = 27, *n* = 1914, countries = 56				
Gross Loss %	SE	Night-time lights growth	0.20	0.01 – 0.40	2.05	0.041*	Travel time to nearest city	-0.12	-0.21 – -0.03	*-2.59*	0.01**
		Community forestry	0.32	0.10 – 0.54	2.80	0.005**	NDC commitment	0.17	0.02 – 0.31	2.20	0.028*
		Restoration effort	0.24	0.05 – 0.43	2.44	0.015*					
	BP	Clumpiness	-0.01	-0.02 – -0.01	-4.15	<0.001***	Clumpiness	-0.05	-0.06 – -0.04	-8.00	<0.001***
		Sediment trapping	0.01	0.00 – 0.01	2.93	0.003**	Sediment trapping	0.01	0.00 – 0.01	2.83	0.005**
		Tidal amplitude	-0.21	-0.28 – -0.14	-6.16	<0.001***	Tidal amplitude	-0.06	-0.12 – -0.00	-2.11	0.035*
		Antecedent SLR	0.09	0.04 – 0.14	3.64	<0.001***					
		Tmin	-0.03	-0.06 – -0.01	-2.82	0.005**					
		Delta	1.90	0.73 – 3.07	3.18	0.001**	Delta	0.98	0.09 – 1.87	2.17	0.03*
		Estuary	1.73	0.59 – 2.86	2.98	0.003**					
		Lagoon	1.70	0.56 – 2.85	2.93	0.003**					
		Open Coast	1.74	0.60 – 2.87	3.00	0.003**					
		ICC = 0.23, R^2m/c = 0.16/0.35, *df* = 27, *n* = 2425, countries = 55					*ICC* = 0.22, R^2m/c = 0.08/0.28, *df* = 27, *n* = 2637, countries = 59				

Net Gain %	SE BP	Restoration effort	0.45	0.07 – 0.84	2.31	0.021*	Night-time lights growth	0.38	0.00 – 0.77	1.96	0.05*
		Indigenous land	-1.52	-2.62 – -0.42	-2.72	0.007**	Community forestry	0.24	0.04 – 0.44	2.31	0.021*
		Clumpiness	-0.12	-0.16 – -0.08	-5.51	<0.001***	Clumpiness	-0.09	-0.13 – -0.05	-4.79	<0.001***
		Antecedent SLR	-0.15	-0.28 – -0.02	-2.27	0.023*	Antecedent SLR	0.11	0.01 – 0.22	2.10	0.036*
		Tmin	-0.20	-0.26 – -0.14	-6.73	<0.001***					
		Delta	2.88	0.08 – 5.68	2.02	0.044*					
		Estuary	3.13	0.45 – 5.80	2.29	0.022*					
		Lagoon	3.22	0.60 – 5.85	2.40	0.016*					
		Open Coast	3.60	0.99 – 6.32	2.69	0.007**					
		ICC = NA, R^2m/c = 0.45/NA, *df* = 27, *n* = 451, countries = 41					*ICC* = 0.14, R^2m/c = 0.14/0.26, *df* = 27, *n* = 743, countries = 49				
Gross Gain %	SE BP	Democracy	-0.87	-1.57 – -0.17	-2.42	0.015*	Community forestry	0.26	0.06 – 0.45	2.62	0.009**
		Clumpiness	-0.09	-0.10 – -0.08	-23.08	<0.001***	Clumpiness	-0.12	-0.14 – -0.10	-14.08	<0.001***
							Tidal amplitude	-0.09	-0.16 – -0.03	-3.03	0.002**
							Antecedent SLR	0.06	0.02 – 0.11	2.71	0.007**
		Tmin	-0.08	-0.10 – -0.05	-5.98	<0.001***	Tmin	-0.06	-0.08 – -0.04	-5.49	<0.001***
		Delta	1.49	0.33 – 2.66	2.52	0.012*	Delta	1.37	0.44 – 2.29	2.89	0.004**
		Estuary	1.27	0.15 – 2.40	2.21	0.027*	Estuary	1.18	0.29 – 2.08	2.59	0.010*
		Lagoon	1.38	0.25 – 2.51	2.38	0.017*	Lagoon	1.14	0.24 – 2.04	2.48	0.013*
		Open Coast	1.51	0.39 – 2.64	2.63	0.008**	Open Coast	1.14	0.25 – 2.03	2.50	0.012*
		ICC = 0.23, R^2m/c = 0.16/0.35, *df* = 27, *n* = 2425, countries = 55					*ICC* = 0.22, R^2m/c = 0.14/0.32, *df* = 27, *n* = 2554, countries = 59				

Supplementary References

1 Bunting, P. Global Mangrove Watch v2.0. UNEP WCMC http://data.unep-wcmc.org/datasets/45 (2018).

2 Worthington, T. A. *et al.* A global biophysical typology of mangroves and its relevance for ecosystem structure and deforestation. *Scientific Reports* **10**, 14652, doi:10.1038/s41598-020-71194-5 (2020).

3 Valiela, I., Bowen, J. L. & York, J. K. Mangrove forests: One of the world's threatened major tropical environments. *Bioscience* **51**, 807-815, doi:10.1641/0006-3568(2001)051[0807:mfootw]2.0.co;2 (2001).

4 Venter, O. *et al.* Sixteen years of change in the global terrestrial human footprint and implications for biodiversity conservation. *Nature Communications* **7**, 12558, doi:10.1038/ncomms12558 (2016).

5 Hochard, J. P., Hamilton, S. & Barbier, E. B. Mangroves shelter coastal economic activity from cyclones. *Proceedings of the National Academy of Sciences* **116**, 12232-12237, doi:10.1073/pnas.1820067116 (2019).

6 Henderson, J. V., Storeygard, A. & Weil, D. N. Measuring Economic Growth from Outer Space. *American Economic Review* **102**, 994-1028, doi:10.1257/aer.102.2.994 (2012).

7 NOAA. Defense Meteorological Program Operational Linescan System (DMSP-OLS) Night-time Lights Time Series version 4 National Geophysical Data Center https://ngdc.noaa.gov/eog/dmsp/downloadV4composites.html (2013).

8 Cinner, J. E. *et al.* Bright spots among the world's coral reefs. *Nature* **535**, 416-419, doi:10.1038/nature18607 (2016).

9 Cinner, J. E. *et al.* Gravity of human impacts mediates coral reef conservation gains. *Proceedings of the National Academy of Sciences* **115**, E6116-E6125, doi:10.1073/pnas.1708001115 (2018).

10 Maire, E. *et al.* How accessible are coral reefs to people? A global assessment based on travel time. *Ecology Letters* **19**, 351-360, doi:10.1111/ele.12577 (2016).

11 Weiss, D. A global map of travel time to cities. 2018).

12 Weiss, D. J. *et al.* A global map of travel time to cities to assess inequalities in accessibility in 2015. *Nature* **553**, 333-336, doi:10.1038/nature25181 (2018).

13 Estrada, A. *et al.* Primates in peril: the significance of Brazil, Madagascar, Indonesia and the Democratic Republic of the Congo for global primate conservation. *Peerj* **6**, doi:10.7717/peerj.4869 (2018).

14 The Observatory of Economic Complexity. Economic Complexity Index 1995-2018 (HS92) https://oec.world/en/rankings/eci/hs4/hs92 (2018).

15 Abman, R. Rule of Law and Avoided Deforestation from Protected Areas. *Ecological Economics* **146**, 282-289, doi:10.1016/j.ecolecon.2017.11.004 (2018).

16 Tørstad, V., Sælen, H. & Bøyum, L. S. The domestic politics of international climate commitments: which factors explain cross-country variation in NDC ambition? *Environmental Research Letters* **15**, 024021, doi:10.1088/1748-9326/ab63e0 (2020).

17 Coppedge, M. *et al.* V-Dem Dataset v10. Varieties of Democracy (V-Dem) Project https://doi.org/10.23696/vdemds20 (2020).

18 Morrison, T. H. *et al.* Political dynamics and governance of World Heritage ecosystems. *Nature Sustainability*, doi:10.1038/s41893-020-0568-8 (2020).

19 Wendling, Z. A., Emerson, J.W., de Sherbinin, A., Esty, D.C., et al. 2020 Environmental Performance Index. Yale Center for Environmental Law & Policy https://epi.yale.edu/ (2020).

20 Frank, C. *et al.* Involvement, knowledge and perception in a natural reserve under participatory management: Mida Creek, Kenya. *Ocean & Coastal Management* **142**, 28-36, doi:https://doi.org/10.1016/j.ocecoaman.2017.03.009 (2017).
21 Estoque, R. C. *et al.* Assessing environmental impacts and change in Myanmar's mangrove ecosystem service value due to deforestation (2000-2014). *Global Change Biology* **24**, 5391-5410, doi:10.1111/gcb.14409 (2018).
22 Santika, T. *et al.* Heterogeneous impacts of community forestry on forest conservation and poverty alleviation: Evidence from Indonesia. *People and Nature* **1**, 204-219, doi:10.1002/pan3.25 (2019).
23 Hajjar, R. *et al.* A global analysis of the social and environmental outcomes of community forests. *Nature Sustainability* **4**, 216-224, doi:10.1038/s41893-020-00633-y (2021).
24 Camacho, L., Gevaña, D., Sabino, L., Ruzol, C., Garcia, J., Camacho, A., Oo, T., Maung, A., Saxena, K., Liang, L., Yiu, E. and Takeuchi, K. Sustainable mangrove rehabilitation: Lessons and insights from community-based management in the Philippines and Myanmar. *APN Science Bulletin* **10**, doi:10.30852/sb.2020.983 (2020).
25 Fa, J. E. *et al.* Importance of Indigenous Peoples' lands for the conservation of Intact Forest Landscapes. *Frontiers in Ecology and the Environment* **n/a**, doi:10.1002/fee.2148 (2020).
26 Brown, B., Fadillah, R., Nurdin, Y., Soulsby, I. & Ahmad, R. CASE STUDY: Community Based Ecological Mangrove Rehabilitation (CBEMR) in Indonesia. *S.A.P.I.EN.S* **7.2** (2014).
27 Lovelock, C. E. & Brown, B. M. Land tenure considerations are key to successful mangrove restoration. *Nature Ecology & Evolution* **3**, 1135-1135, doi:10.1038/s41559-019-0942-y (2019).
28 Garnett, S. T. *et al.* A spatial overview of the global importance of Indigenous lands for conservation. *Nature Sustainability* **1**, 369-374, doi:10.1038/s41893-018-0100-6 (2018).
29 Herr, D. & Landis, E. (IUCN and TNC, Gland, Switzerland and Washington, DC, USA, 2016).
30 Duarte, C. M. *et al.* Rebuilding marine life. *Nature* **580**, 39-51, doi:10.1038/s41586-020-2146-7 (2020).
31 Turschwell, M. P. *et al.* Multi-scale estimation of the effects of pressures and drivers on mangrove forest loss globally. *Biological Conservation* **247**, 108637, doi:10.1016/j.biocon.2020.108637 (2020).
32 Gill, D. A. *et al.* Capacity shortfalls hinder the performance of marine protected areas globally. *Nature* **543**, 665-669, doi:10.1038/nature21708 (2017).
33 Davidson, N. C. *et al.* Trends in the ecological character of the world's wetlands. *Marine and Freshwater Research* **71**, 127-138, doi:10.1071/mf18329 (2020).
34 Balke, T. & Friess, D. A. Geomorphic knowledge for mangrove restoration: a pan-tropical categorization. *Earth Surface Processes and Landforms* **41**, 231-239, doi:https://doi.org/10.1002/esp.3841 (2016).
35 Thomas, N. *et al.* Distribution and drivers of global mangrove forest change, 1996–2010. *PLOS ONE* **12**, e0179302, doi:10.1371/journal.pone.0179302 (2017).
36 Twilley, R. R., Castañeda-Moya, E., Rivera-Monroy, V. H. & Rovai, A. in *Mangrove Ecosystems: A Global Biogeographic Perspective: Structure, Function, and Services* (eds Victor H. Rivera-Monroy, Shing Yip Lee, Erik Kristensen, & Robert R. Twilley) (Springer International Publishing, 2017).

37 van Bijsterveldt, C. E. J. *et al.* How to restore mangroves for greenbelt creation along eroding coasts with abandoned aquaculture ponds. *Estuarine, Coastal and Shelf Science*, 106576, doi:https://doi.org/10.1016/j.ecss.2019.106576 (2020).
38 Maynard, J. *et al.* Mangrove cover change between 1996 and 2016 near river-ocean outlets: A global analysis to identify priority areas for conservation. (World Wildlife Fund, Washington D.C., 2019).
39 Grill, G. *et al.* Mapping the world's free-flowing rivers. *Nature* **569**, 215-+, doi:10.1038/s41586-019-1111-9 (2019).
40 Bryan-Brown, D. N. *et al.* Global trends in mangrove forest fragmentation. *Scientific Reports* **10**, 7117, doi:10.1038/s41598-020-63880-1 (2020).
41 Simard, M. *et al.* Mangrove canopy height globally related to precipitation, temperature and cyclone frequency. *Nature Geoscience* **12**, 40-45, doi:10.1038/s41561-018-0279-1 (2019).
42 Rovai, A. S. *et al.* Scaling mangrove aboveground biomass from site-level to continental-scale. *Global Ecology and Biogeography* **25**, 286-298, doi:10.1111/geb.12409 (2016).
43 Stammer, D. *et al.* Accuracy assessment of global barotropic ocean tide models. *Reviews of Geophysics* **52**, 243-282, doi:10.1002/2014RG000450 (2014).
44 Noveltis, Legos & CLS. FES2014. Aviso+ with support from Cnes https://www.aviso.altimetry.fr/ (2014).
45 Lovelock, C. E. *et al.* The vulnerability of Indo-Pacific mangrove forests to sea-level rise. *Nature* **526**, 559-563, doi:10.1038/nature15538 (2015).
46 Kirwan, M. L. & Megonigal, J. P. Tidal wetland stability in the face of human impacts and sea-level rise. *Nature* **504**, 53-60, doi:10.1038/nature12856 (2013).
47 Duke, N. C., Field, C., Mackenzie, J. R., Meynecke, J.-O. & Wood, A. L. Rainfall and its possible hysteresis effect on the proportional cover of tropical tidal-wetland mangroves and saltmarsh–saltpans. *Marine and Freshwater Research* **70**, 1047-1055, doi:https://doi.org/10.1071/MF18321 (2019).
48 Lovelock, C. E., Feller, I. C., Reef, R., Hickey, S. & Ball, M. C. Mangrove dieback during fluctuating sea levels. *Scientific Reports* **7**, 1680, doi:10.1038/s41598-017-01927-6 (2017).
49 Hickey, S. M. *et al.* ENSO feedback drives variations in dieback at a marginal mangrove site. *Scientific Reports* **11**, 8130, doi:10.1038/s41598-021-87341-5 (2021).
50 Legeais, J. F. *et al.* An improved and homogeneous altimeter sea level record from the ESA Climate Change Initiative. *Earth Syst. Sci. Data* **10**, 281-301, doi:10.5194/essd-10-281-2018 (2018).
51 Osland, M. J. *et al.* Climatic controls on the global distribution, abundance, and species richness of mangrove forests. *Ecological Monographs* **87**, 341-359, doi:10.1002/ecm.1248 (2017).
52 Duke, N. C. *et al.* Large-scale dieback of mangroves in Australia's Gulf of Carpentaria: a severe ecosystem response, coincidental with an unusually extreme weather event. *Marine and Freshwater Research* **68**, 1816-1829, doi:10.1071/MF16322 (2017).
53 Vicente-Serrano, S. M. & Beguería, S. SPEIbase v.2.6. http://hdl.handle.net/10261/202305 (2020).
54 Vicente-Serrano, S. M., Beguería, S. & López-Moreno, J. I. A Multi-scalar drought index sensitive to global warming: The Standardized Precipitation Evapotranspiration Index – SPEI. *Journal of Climate* **23**, 1696-1718, doi:10.1175/2009JCLI2909.1 (2010).

55 Vicente-Serrano, S. M., Beguería, S., López-Moreno, J. I., Angulo, M. & El Kenawy, A. A global 0.5° gridded dataset (1901-2006) of a multiscalar drought index considering the joint effects of precipitation and temperature. *Journal of Hydrometeorology* **11**, 1033-1043, doi:10.1175/2010JHM1224.1 (2010).
56 Villamayor, B. M. R., Rollon, R. N., Samson, M. S., Albano, G. M. G. & Primavera, J. H. Impact of Haiyan on Philippine mangroves: Implications to the fate of the widespread monospecific Rhizophora plantations against strong typhoons. *Ocean & Coastal Management* **132**, 1-14, doi:10.1016/j.ocecoaman.2016.07.011 (2016).
57 Sippo, J. Z., Lovelock, C. E., Santos, I. R., Sanders, C. J. & Maher, D. T. Mangrove mortality in a changing climate: An overview. *Estuarine, Coastal and Shelf Science* **215**, 241-249, doi:10.1016/j.ecss.2018.10.011 (2018).
58 NOAA. International Best Track Archive for Climate Stewardship (IBTrACS) version 4. https://www.ncdc.noaa.gov/ibtracs/ (2020).
59 Ross, M. S., Ruiz, P.L., Sah, J.P., Hanan, E.J. Chilling damage in a changing climate in coastal landscapes of the subtropical zone: a case study from south Florida. *Global Change Biology* **15**, 1817–1832, doi:10.1111/j.1365-2486.2009.01900.x (2009).
60 Osland, M. J., Day, R. H., Larriviere, J. C. & A.S., F. Aboveground Allometric Models for Freeze-Affected Black Mangroves (Avicennia germinans): Equations for a Climate Sensitive Mangrove-Marsh Ecotone. *PLoS ONE* **9(6)**, e99604, doi:10.1371/journal.pone.0099604 (2014).
61 Chen, L. *et al.* Mangrove species' responses to winter air temperature extremes in China. *Ecosphere* **8**, e01865, doi:https://doi.org/10.1002/ecs2.1865 (2017).
62 Osland M.J., D. R. H., Larriviere J.C., From A.S. Aboveground Allometric Models for Freeze-Affected Black Mangroves (Avicennia germinans): Equations for a Climate Sensitive Mangrove-Marsh Ecotone. *PLoS ONE* **9(6)**, e99604, doi:10.1371/journal.pone.0099604 (2014).
63 Fick, S. E. & Hijmans, R. J. WorldClim 2: new 1km spatial resolution climate surfaces for global land areas. *International Journal of Climatology* **37 (12)**, 4302-4315 (2017).
64 Harris, I., Jones, P. D., Osborn, T. J. & Lister, D. H. Updated high-resolution grids of monthly climatic observations - the CRU TS3.10 Dataset. *International Journal of Climatology* **34**, 623-642, doi:10.1002/joc.3711 (2014).
