# Supplementary material for: Drivers of global mangrove loss and gain in social-ecological systems
Source: Nat Commun. 2022 Oct 26;13:6373. doi: 10.1038/s41467-022-33962-x (PMC9606261; doi:10.1038/s41467-022-33962-x)
Supplement: Supplementary file 2 — Description of Additional Supplementary Files [file 41467_2022_33962_MOESM2_ESM.pdf]

File Name: Supplementary Data 1
Description: Sample size per country in the full dataset and models, and national extent of mangrove cover in 1996, 2007 and 2016
