# Supplementary material for: Drivers of global mangrove loss and gain in social-ecological systems
Source: Nat Commun. 2022 Oct 26;13:6373. doi: 10.1038/s41467-022-33962-x (PMC9606261; doi:10.1038/s41467-022-33962-x)
Supplement: Supplementary file 4 — Reporting Summary [file 41467_2022_33962_MOESM4_ESM.pdf]

nature portfolio

Corresponding author(s): Valerie Hagger

Last updated by author(s): Aug 22, 2022

Reporting Summary

Nature Portfolio wishes to improve the reproducibility of the work that we publish. This form provides structure for consistency and transparency in reporting. For further information on Nature Portfolio policies, see our Editorial Policies and the Editorial Policy Checklist.

Statistics

For all statistical analyses, confirm that the following items are present in the figure legend, table legend, main text, or Methods section.

n/a	Confirmed	
☐	☒	The exact sample size (*n*) for each experimental group/condition, given as a discrete number and unit of measurement
☒	☐	A statement on whether measurements were taken from distinct samples or whether the same sample was measured repeatedly
☐	☒	The statistical test(s) used AND whether they are one- or two-sided<br>*Only common tests should be described solely by name; describe more complex techniques in the Methods section.*
☐	☒	A description of all covariates tested
☐	☒	A description of any assumptions or corrections, such as tests of normality and adjustment for multiple comparisons
☐	☒	A full description of the statistical parameters including central tendency (e.g. means) or other basic estimates (e.g. regression coefficient) AND variation (e.g. standard deviation) or associated estimates of uncertainty (e.g. confidence intervals)
☐	☒	For null hypothesis testing, the test statistic (e.g. *F*, *t*, *r*) with confidence intervals, effect sizes, degrees of freedom and *P* value noted<br>*Give P values as exact values whenever suitable.*
☒	☐	For Bayesian analysis, information on the choice of priors and Markov chain Monte Carlo settings
☐	☒	For hierarchical and complex designs, identification of the appropriate level for tests and full reporting of outcomes
☐	☒	Estimates of effect sizes (e.g. Cohen's *d*, Pearson's *r*), indicating how they were calculated

Our web collection on statistics for biologists contains articles on many of the points above.

Software and code

Policy information about availability of computer code

Data collection	R 4.0.2, R 3.6.3, Raster package v. 3.5.2, landscapemetrics package v. 1.5-0, ncdf4 package v. 1.19, sf package v 1.0-5, PuTTY (https://www.putty.org/) to send R code to The University of Queensland high performance computers, ArcGIS Desktop 10.8
Data analysis	R 4.0.2, ArcGIS Desktop 10.8, lme4 package v. 1.1-27.1, spatialreg package v. 1.1-5, spdep package v. 1.1-5, ncf package v. 1.2-9.<br>R code developed for the driver and hotspot analysis, figure production, and for the calculation of community forestry, NDC and mangrove restoration indices are available in UQ eSpace [https://doi.org/10.48610/233461e]. R code can be used together with the published datasets (see 'Data availability') to recalculate the study results.

For manuscripts utilizing custom algorithms or software that are central to the research but not yet described in published literature, software must be made available to editors and reviewers. We strongly encourage code deposition in a community repository (e.g. GitHub). See the Nature Portfolio guidelines for submitting code & software for further information.

Data

Policy information about availability of data

All manuscripts must include a data availability statement. This statement should provide the following information, where applicable:

- Accession codes, unique identifiers, or web links for publicly available datasets
- A description of any restrictions on data availability
- For clinical datasets or third party data, please ensure that the statement adheres to our policy

The datasets generated in this study for the analysis of drivers and hotspots of mangrove loss and gain, and national datasets on community forestry, NDCs, and mangrove restoration have been deposited in UQ eSpace [https://doi.org/10.48610/233461e]. The number of sampling units per country used in the analysis and the national extent of mangrove cover in 1996, 2007 and 2016 are provided in Supplementary Data 1.

Original data used in the study for the calculation of variables are available as follows: Global Mangrove Watch from the Ocean Data Viewer [http://data.unep-wcmc.org/datasets/45], global biophysical mangrove typology from the Ocean Data Viewer [https://data.unep-wcmc.org/datasets/48], country boundaries from GADM data [https://gadm.org/data.html], Exclusive Economic Zones from Marine Regions [https://marineregions.org/downloads.php], Nighttime Lights Time Series from the National Geophysical Data Center [https://ngdc.noaa.gov/eog/dmsp/downloadV4composites.html], Economic Complexity Index from The Observatory of Economic Complexity [https://oec.world/en/rankings/eci/hs4/hs92], Varieties of Democracy from the V-Dem Dataset [https://doi.org/10.23696/vdemds20], community forestry projects from the ICCA Registry [https://www.iccaregistry.org/] and REDD+ projects database [http://www.reddprojectsdatabase.org], National Determined Contributions from the NDC registry [https://www4.unfccc.int/sites/ndcstaging/Pages/Home.aspx], Ramsar sites from the Ramsar Sites Information Service [https://rsis.ramsar.org/], Environmental Sustainability Index from the Socioeconomic Data and Applications Center [https://doi.org/10.7927/H40V89R6], marine protected area management data in Supplementary Data 1 under doi:10.1038/nature21708 [https://doi.org/10.1038/nature21708], Sediment Trapping Index from the global free-flowing rivers dataset [https://doi.org/10.6084/m9.figshare.7688801], river networks from the HydroSHEDS database [https://www.hydrosheds.org/hydrosheds-core-downloads], tidal amplitude in FES2014 from the Aviso+ Cnes Data Center [https://www.aviso.altimetry.fr/], sea-level rise in the global sea level ECV product from the CCI Open Data Portal [http://doi.org/10.5270/esa-sea_level_cci-MSLA-1993_2015-v_2.0-201612], SPEI from SPEIbase [https://digital.csic.es/handle/10261/202305], tropical storms from the IBTrACS database [https://www.ncei.noaa.gov/products/international-best-track-archive?name=ib-v4-access], and bioclimatic variables from the WorldClim database [https://www.worldclim.org/data/worldclim21.html].

Field-specific reporting

Please select the one below that is the best fit for your research. If you are not sure, read the appropriate sections before making your selection.

☐ Life sciences ☐ Behavioural & social sciences ☒ Ecological, evolutionary & environmental sciences

For a reference copy of the document with all sections, see nature.com/documents/nr-reporting-summary-flat.pdf

Ecological, evolutionary & environmental sciences study design

All studies must disclose on these points even when the disclosure is negative.

Study description	A global analysis of the drivers of mangrove cover losses and gains in complex social-ecological systems over varying spatial and temporal scales. Hierarchical statistical modelling was used to quantify the relationship between four measures of mangrove cover change (% net loss, % gross loss, % net gain, % gross gain) across landscape geomorphic units (n = 4394 across 108 countries) and two time periods (1996-2007 and 2007-2016), and eight socioeconomic and seven biophysical variables to consider national- and landscape-level variability. Country was included as a varying-intercept and travel time to the nearest city and nighttime lights growth as varying-slopes in the eight models. Subsetting of the dataset was required to remove experimental units that were smaller than 1 ha and had no change in mangrove cover and separate units with loss (negative change) and gain (positive change). Experimental units with missing data from the global datasets were also removed, resulting in the following number of units per model: % net loss 1996-2007 (n = 2004), % net loss 2007-2016 (n = 1914), % gross loss 1996-2007 (n = 2425), % gross loss 2007-2016 (n = 2637), % net gain 1996-2007 (n = 451), % net gain 2007-2016 (n = 743), % gross gain 1996-2007 (n = 2341), % gross gain 2007-2016 (n = 2554). Mangrove geomorphic units were sampled across two time periods. Therefore there was replication of the same location sampled twice. However, each time period was analysed in a separate model, therefore there was no replication in the model. Also in some instances geomorphic units changed from loss to no change to gain across the time periods. Thus, the composition of geomorphic units were not identical across the time periods.
Research sample	The research sample was chosen to represent global mangrove extent using remote sensing data. The Global Mangrove Watch (GMW) dataset (Bunting 2018), a high resolution, global time-series on mangrove cover from 1996-2016, was used to calculate mangrove cover change between 1996 and 2007, and 2007 and 2016 across landscape mangrove geomorphic units (n = 4329) (Worthington et al. 2020). The GMW produces 25 m spatial resolution maps of global mangrove extent using Japanese L-band Synthetic Aperture Radar (SAR) data and Landsat sensor data.<br>We undertook a synthesis of national policies and actions to derive new global datasets for conservation factors at the country-level of all mangrove-holding nations. Existing global datasets were used for estimating socio-economic and biophysical variables at mangrove geomorphic units across all mangrove-holding nations.
Sampling strategy	No sample size calculation was performed because the research sample included the global extent of mangrove cover mapped. Sample sizes were driven by the assessing mangrove cover changes across mangrove geomorphic units (response variables). A global biophysical typology of mangroves was used to delineate global mangrove cover into 4394 geomorphic units. Explanatory variables were assessed at either the national- or landscape-level for mangrove geomorphic units. The size of mangrove geomorphic units varied from 1-2640 km2 and the number of mangrove geomorphic units per country varied from 1-612. To control for the effect of size of the mangrove geomorphic unit on mangrove cover change, percent measures (loss or gain relative to the area at the start of the time period) were used as the response variables. In our dataset, some countries only have one geomorphic unit (12 countries) whilst some countries have many (e.g. Australia and Indonesia have 519 and 822 units, respectively). Multi-level modelling was used to minimise extreme estimates for countries with small sample sizes by allowing estimates to be closer to the complete pooling approach (Gelman and Hill, 2007).<br>Because of the unavailability of socio-economic indicators for many countries, and no change in mangrove cover at many mangrove geomorphic units, and the requirement to remove NAs and zeros from the dataset, as well as geomorphic units less than 100 ha to avoid bias is our results, the research sample was reduced to 3134 units across 95 countries. The subsetted dataset comprised 89% and 90% of the 2016 global mangrove extent for the two decades; therefore our modelling represents drivers of the majority of mangrove holdings. The hotspot analysis was done on the full dataset (n=4329), representing all the mangrove-holdings across the world detected by remote sensing.
Data collection	The GMW dataset and existing global datasets for the socio-economic and biophysical variables were downloaded from publicly-available data repositories by Tom Worthington and Valerie Hagger.<br>Valerie Hagger produced the new global datasets for conservation factors through systematic literature review searches and

	searches on online databases.
Timing and spatial scale	The GMW time series is currently available from 1996-2016, therefore we choose our time periods to assess changes in the drivers of mangrove loss and gain to match the available dataset on mangrove mapping. This is relevant because mangrove losses were estimated to be significant by the end of the 1990s, but since then rates of loss have declined, so we assessed change across the two decades of the GMW data (1996-2007 and 2007-2016). There is no GMW time series for 2006, therefore 2007 was used. Data collection on explanatory variables commenced in March 2020 and ended in July 2021. Where possible, explanatory variables specific to the time-period and the mangrove geomorphic scale were calculated. The spatial scale was global covering 108 mangrove-holding countries in tropical and subtropical regions.
Data exclusions	Two datasets were excluded from the statistical modelling due to collinearity with other variables; Environmental Performance Index (Yale Center for Environmental Law and Policy, 2005) and Marine Protected Area staff capacity (Gill et al. 2017).<br>Experimental units that were smaller than 1 ha, had no change in mangrove cover, and had missing data from the global datasets were removed from statistical analysis. This exclusion criteria was pre-established due to the following rationale: (1) a minimum mapping unit of 1 ha is recommended for reliable results in using the GMW mapping, (2) logging of the response variable was required to meet the assumptions of normal distribution for linear modelling, and (3) removing NAs from the dataset for analysis.<br>During the data exploration it was revealed that small mangrove geomorphic units typically displayed very high percent losses or gains, therefore we also removed experimental units smaller than 100 ha to avoid bias towards small geomorphic units.
Reproducibility	Initially nine mangrove cover change indicators were included in the statisical modelling which helped to verify reproducibility of the results by running and comparing models using different measures of mangrove cover change and resolving issues with model fit. Extraction of variables from global datasets was conducted with R code, some via the the UQ high performance computers, which allows reproducibility of variable generation.
Randomization	Sampling was undertaken across mangrove geomorphic units (classification of mangrove areas as delta, estuary, lagoon or open coast) across the global extent of mangrove cover. Randomisation was not relevant, because we aimed to sample all mangrove geomorphic units across mangrove-holding countries.
Blinding	Blinding was not relevant to our study, because it did not involve study participants; the study was a desktop-based global analysis using publicly-available datasets and generating new global datasets based on review of literature and databases.

Did the study involve field work? ☐ Yes ☒ No

Reporting for specific materials, systems and methods

We require information from authors about some types of materials, experimental systems and methods used in many studies. Here, indicate whether each material, system or method listed is relevant to your study. If you are not sure if a list item applies to your research, read the appropriate section before selecting a response.

Materials & experimental systems

n/a	Involved in the study	
☒	☐	Antibodies
☒	☐	Eukaryotic cell lines
☒	☐	Palaeontology and archaeology
☒	☐	Animals and other organisms
☒	☐	Human research participants
☒	☐	Clinical data
☒	☐	Dual use research of concern

Methods

n/a	Involved in the study	
☒	☐	ChIP-seq
☒	☐	Flow cytometry
☒	☐	MRI-based neuroimaging
